# Role of the IL-33/ST2 axis in cardiovascular disease: A systematic review and meta-analysis

**DOI:** 10.1371/journal.pone.0259026

**Published:** 2021-11-01

**Authors:** Yuan Sun, Holly Pavey, Ian Wilkinson, Marie Fisk

**Affiliations:** Division of Experimental Medicine and Immunotherapeutics, Department of Medicine, University of Cambridge, Cambridge, United Kingdom; Medizinische Universitat Graz, AUSTRIA

## Abstract

Interleukin (IL)-33 and its unique receptor, ST2, play a pivotal role in the immune response to infection and stress. However, there have been conflicting reports of the role of IL-33 in cardiovascular disease (CVD) and the potential of this axis in differentiating CVD patients and controls and with CVD disease severity, remains unclear.

**Aims:**

1) To quantify differences in circulating IL-33 and/or sST2 levels between CVD patients versus controls. 2) Determine association of these biomarkers with mortality in CVD and community cohorts.

**Methods and results:**

Using Pubmed/MEDLINE, Web of Science, Prospero and Cochrane databases, systematic review of studies published on IL-33 and/or sST2 levels in patients with CVD (heart failure, acute coronary syndrome, atrial fibrillation, stroke, coronary artery disease and hypertension) vs controls, and in cohorts of each CVD subtype was performed. Pooled standardised mean difference (SMD) of biomarker levels between CVD-cases versus controls and hazard ratios (HRs) for risk of mortality during follow-up in CVD patients, were assessed by random effects meta-analyses. Heterogeneity was evaluated with random-effects meta-regressions. From 1071 studies screened, 77 were meta-analysed. IL-33 levels were lower in HF and CAD patients vs controls, however levels were higher in stroke patients compared controls [Meta-SMD 1.455, 95% CI 0.372–2.537; p = 0.008, I^2^ = 97.645]. Soluble ST2 had a stronger association with risk of all-cause mortality in ACS (Meta-multivariate HR 2.207, 95% CI 1.160–4.198; p = 0.016, I^2^ = 95.661) than risk of all-cause mortality in HF (Meta-multivariate HR 1.425, 95% CI 1.268–1.601; p<0.0001, I^2^ = 92.276). There were insufficient data to examine the association of IL-33 with clinical outcomes in CVD.

**Conclusions:**

IL-33 and sST2 levels differ between CVD patients and controls. Higher levels of sST2 are associated with increased mortality in individuals with CVD. Further study of IL-33/ST2 in cardiovascular studies is essential to progress diagnostic and therapeutic advances related to IL-33/ST2 signalling.

## Introduction

Cardiovascular diseases (CVDs) are the leading causes of death and morbidity worldwide. In the UK alone, CVDs caused 167,116 (27.1%) of all deaths in 2018 [1]. Currently there is an unmet need to improve identification of patients with CVD, enhance prognostic prediction of patients who are at risk of poor outcomes and improve understanding of the underlying pathophysiology of CVD to enable development of novel treatment targets.

Inflammatory pathways play an important role in atherosclerosis, and an improved understanding of the signalling molecules involved has translated to promising therapeutics such as the CANTOS trial targeting Interleukin-1β [2]. Interleukin-33 (IL-33) is another member of the Interleukin-1 family of cytokines that has emerged as a target of interest in a variety of inflammatory conditions including asthma, chronic obstructive pulmonary disease, and most recently Coronavirus disease 2019 (COVID-19) [3–5]. Despite these advances, pre-clinical studies on the role of IL-33 in cardiovascular health have yielded contrasting data, suggesting both cardioprotective and detrimental effects of IL-33 [6].

Previous studies have elucidated the biological function of IL-33 as constitutively expressed and stored in complex with chromatin in the nucleus of epithelial cells and vascular endothelium [7, 8]. In response to cellular injury or necrosis, the chromatin-IL-33 complex is released into the extracellular space as an alarmin signal. When bound to ST2L (transmembrane ligand of IL-33’s unique receptor, ST2) on target cell membranes, it triggers an intracellular signalling cascade, augmented by the presence of histones [9]. IL-33 induces varied immune cells and can upregulate the release of cytokines such as IL-6 and IL-8 [10]. IL-33’s unique receptor, ST2, exists in a transmembrane (ST2L) and soluble form (sST2). Major sources of ST2L and sST2 include endothelial cells of the aorta and coronary arteries as well as immune cells such as T cells [11, 12]. The transmembrane form of ST2 enables IL-33’s signalling activity, whilst sST2 acts as a decoy receptor binding IL-33, to dampen its effects.

Soluble ST2 has been evaluated in a number of clinical CVD studies and is a Food and Drug Administration (FDA) approved prognostic biomarker of mortality in chronic heart failure patients [13]. Previous meta-analyses have shown that sST2 has diagnostic value for heart failure and is prognostic for all-cause mortality in heart failure, coronary artery disease and following aortic valve replacement [14–19]. However, there is a paucity of clinical studies on IL-33, which is likely due to difficultly measuring circulating levels [20].

To our knowledge, no previous meta-analysis has examined IL-33 itself, or with sST2 as biomarkers across the spectrum of CVDs. In this systematic review and meta-analysis, we sought to systematically evaluate data to establish the clinical significance of the IL-33/ST2 axis in CVD. Firstly, to determine the value of circulating IL-33 and/or sST2 levels in differentiating people with CVDs versus those without. Secondly, to determine the association of IL-33 and/or sST2 biomarker levels with clinical outcomes of mortality or MACE (composite endpoint of death or adverse cardiovascular event) in CVD or community cohorts.

## Materials and methods

### Search strategy

Before beginning the search process, we registered this systematic review and meta-analysis on Prospero (https://www.crd.york.ac.uk/prospero/ - (CRD42020168206).

MF devised the study question and design and contributed to data collection and analysis, and co-authored the final manuscript. IBW contributed to study design and manuscript writing. HP contributed to statistical analysis. YS undertook data collection, data analysis and statistical analysis and wrote the original draft of the manuscript.

The authors carried out independent searches in Pubmed (including MEDLINE), Web of Science, Cochrane Library and Prospero databases in September 2020. The search terms were one of IL-33/Interleukin-33/ST2 combined with one of cardiovascular disease, stroke, myocardial infarction, heart failure, coronary disease, ischaemic heart disease and hypertension. The same search terms were entered into each database.

During primary screening of articles, the title and abstract were assessed for relevance to the topic, English language, full text availability and inclusion/exclusion criteria. All datasets included in this systematic review and meta-analysis were *in vivo* human studies that involved measurement of plasma or serum levels of IL-33 or sST2 as a continuous variable (not categorical) and a subtype of CVD (based on clinical diagnosis and/or supporting clinical test data of this diagnosis). Full inclusion/exclusion criteria are available in S1 File. Studies were undertaken in countries across the world, with China being the largest single source. Meta-analyses were performed for CVDs versus controls based on CVD search terms listed above. Studies were categorised into acute coronary syndrome (ACS, including unstable angina and myocardial infarction), coronary artery disease (CAD, including stable angina), atrial fibrillation (AF), systemic hypertension, acute heart failure (defined as hospital admission with decompensation or new hospital diagnosis) or chronic heart failure (CHF). Individual meta-analysis stratified by these distinct CVD subtypes vs controls were performed if there were ≥2 eligible studies. The association of IL-33 and/or sST2 biomarker levels with clinical outcomes of all-cause mortality or MACE (composite endpoint of death or adverse cardiovascular event) were evaluated in CVD as well as community population studies.

### Data extraction and quality assessment

The authors independently used the PRISMA statement as the basis for extracting and recording data from eligible articles. Data extracted includes circulating biomarker levels and associated standard deviation or confidence intervals, inclusion and exclusion criteria of the study, study type, sample size, hazard ratios, follow-up period, age and outcomes. Where median biomarker levels and quartile values were reported, they were converted to mean and standard deviations with the method reported by Wan *et al*. 2014 to enable standardisation across studies [21]. Studies reporting data that could not be converted to mean and standard deviation were not included in analyses where the outcome was standardised mean difference. Full data extracted, including adjustment factors of multivariate HR analysis are available in (S3 Table in S3 File). Disagreements over the eligibility of a study and its data were resolved through discussion.

Study quality was assessed using a modified version of the Quality Assessment of Diagnostic Accuracy Studies 2 (QUADAS-2) criteria. Full criteria are available in S2 File.

### Statistical analysis

The primary outcomes were weighted pooled standardised mean difference (SMD) of biomarker levels comparing cases vs controls (Meta-SMD) and weighted pooled hazard ratios (Meta-HR) for occurrence of all-cause mortality, cardiovascular death and MACE during follow up calculated using random effects models. Only studies presenting univariate or multivariate HRs per (log) unit increase in biomarker levels were accepted for Meta-HR.

Data selected for meta-analysis were displayed in forest plots showing the SMDs or HRs of individual studies and the overall Meta-value. Heterogeneity was assessed using I^2^ statistics and where possible, meta-regression was carried out to assess the effect of cohort age (mean or median), length of follow up (mean or median), publication year, biomarker assay type (Presage ST2 or not) [13]. Statistical analysis was carried out using Comprehensive Meta-Analysis 3.0 software.

## Results and discussion

**Fig 1:**
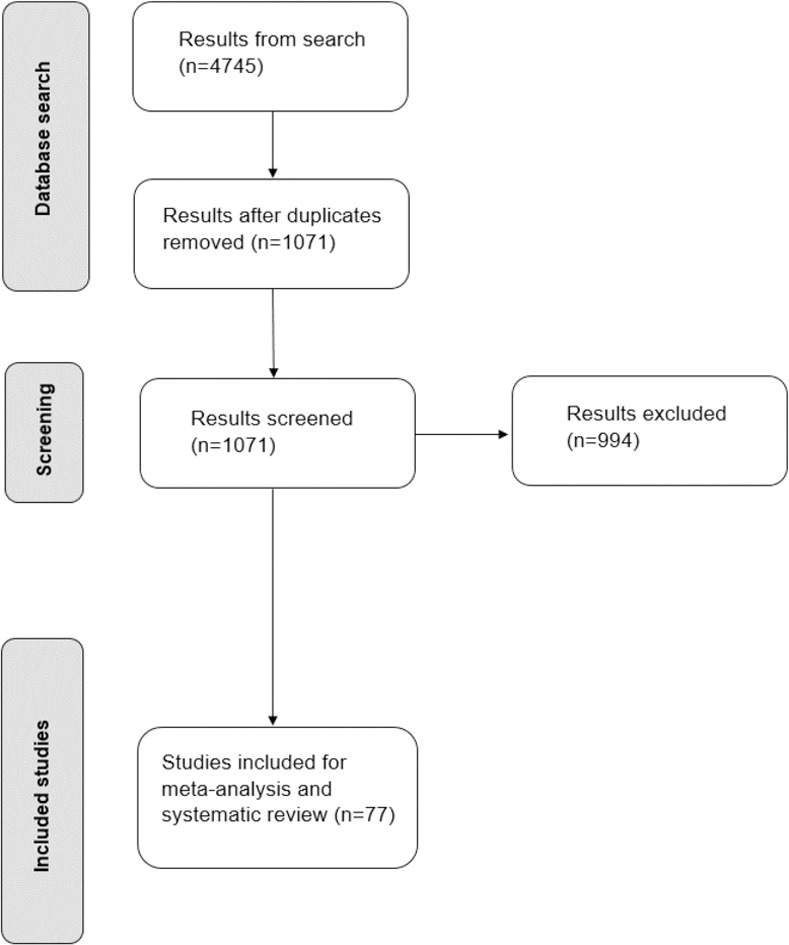
Flow chart of search strategy. Summary of search strategy and eligible study selection.

The flow diagram summary of search results is shown in Fig 1. 4745 articles were identified, and, after duplicates were removed and screening was carried out, remaining studies were systematically screened, yielding 77 studies (62075 participants) for meta-analysis and systematic review. The vast majority of included studies assessed participants between 60–80 years old and heart failure was the most reported disease type (47% of studies). A full table detailing the studies can be found in S3 File.

### IL-33 and sST2 levels in CVD patients versus controls

#### IL-33

**Fig 2:**
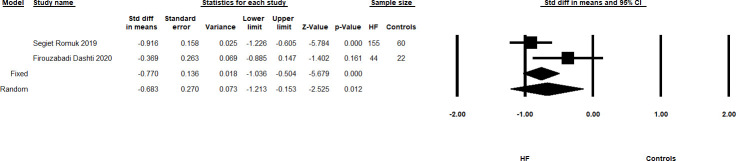
Summary forest plots showing the Meta-SMD and 95% CI of IL-33 levels in HF patients and healthy controls. The Meta-SMD in the random effects model is shown by the black diamond at the bottom. The vertical line at 0.00 is the border for significance.

In analysis of two CAD studies with a total sample size of 156 subjects, patients had lower IL-33 levels than controls, Meta-SMD of -0.972, 95% CI -1.307-(-0.638); p<0.0001, I^2^ = 0.0. Additionally, analysis of two HF studies with 281 total subjects showed that patients had lower IL-33 levels than controls, Meta-SMD of -0.683, 95% CI -1.213-(-0.153); p = 0.012, I^2^ = 68.467 (Fig 2). ACS patients also had lower IL-33 levels compared to controls (four studies with total sample size of 331), although this did not reach statistical significance: Meta-SMD of -1.373, 95% CI -2.978–0.231; p = 0.093, I^2^ = 97.379.

**Fig 3:**
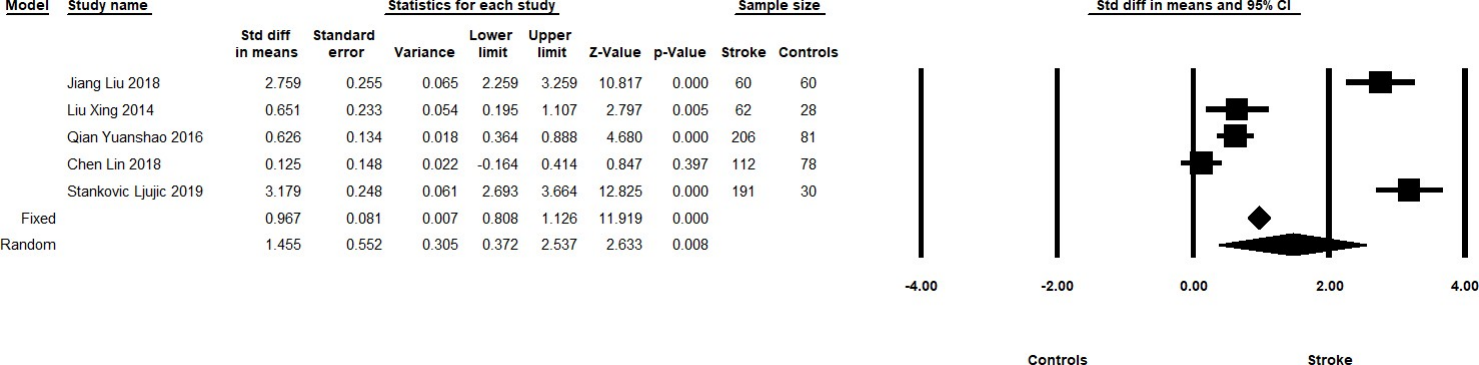
Summary forest plots showing the Meta-SMD and 95% CI of IL-33 levels in stroke patients and healthy controls. The Meta-SMD in the random effects model is shown by the black diamond at the bottom. The vertical line at 0.00 is the border for significance.

Five studies reported (908 total subjects, sample size ranging from 90–287) IL-33 levels in acute ischaemic stroke patients vs controls. Stroke patients had consistently higher IL-33 levels than healthy controls [Meta-SMD 1.455, 95% CI 0.372–2.537; p = 0.008, I^2^ = 97.645] (Fig 3). Two studies (449 subjects) showed that stroke patients who had favourable outcomes (Barthel Index score above 85 or 90 respectively at 3 months and 1 year after admission respectively) had higher baseline IL-33 levels than those who did not: Meta-SMD 0.564, 95% CI 0.356–0.772; p<0.0001, I^2^ = 0.0 [22, 23].

There was no difference in IL-33 levels between hypertension patients and controls (three studies with total sample size of 710): Meta-SMD of -0.024, 95% CI -0.443–0.395; p = 0.912, I^2^ = 81.874.

There were no clinical studies to evaluate the association of IL-33 with clinical outcomes based on the study’s inclusion/exclusion criteria.

#### sST2

Analysis of two CAD studies (408 subjects) showed patients had no difference in levels of sST2 compared with controls: Meta-SMD of 0.033 [95% CI -0.197–0.264; p = 0.778, I^2^ = 16.127].

Only one study across the meta-analysis of all CVD subtypes, Demyanets *et al*. 2014 in CAD, reported patients having lower sST2 levels than controls [24]. Four studies with a total of 470 subjects reported that HF patients had higher sST2 levels than controls: Meta-SMD of 2.178 [95% CI 0.653–3.704; p = 0.005, I^2^ = 96.939].

**Fig 4:**
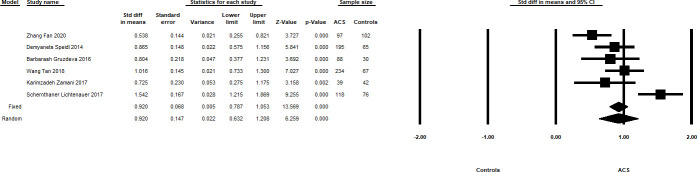
Summary forest plots showing the Meta-SMD and 95% CI of sST2 levels in ACS patients and healthy controls. The Meta-HR in the random effects model is shown by the black diamond at the bottom. The vertical line at 1 is the border for significance.

Meta-analyses of sST2 performed within ACS vs controls (total sample size of 1153) are shown in Fig 4, where ACS patients had higher levels of sST2 compared with controls: Meta-SMD of 0.92 [95% CI 0.632–1.208; p<0.0001, I^2^ = 77.797].

**Fig 5:**
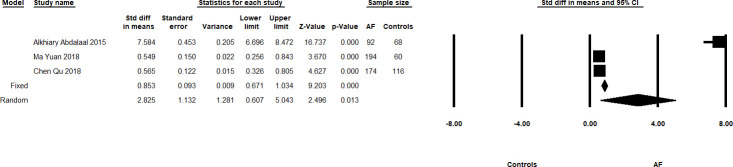
Summary forest plots showing the Meta-SMD and 95% CI of sST2 levels in AF patients and healthy controls. The Meta-HR in the random effects model is shown by the black diamond at the bottom. The vertical line at 1 is the border for significance.

Acute ischaemic stroke patients also had higher sST2 levels compared to controls, reported from two studies with a sample size of 190 and 221, although this did not reach statistical significance [Meta-SMD 3.96, 95% CI -0.839–8.760; p = 0.106, I^2^ = 99.334]. Additionally, AF patients also had higher sST2 levels than controls (Fig 5, 704 subjects from three studies): Meta-SMD of 2.825 [95% CI 0.607–5.043; p = 0.013, I^2^ = 99.132].

### Association of sST2 levels and clinical outcomes in CVD and community cohorts

For CAD, three studies following 4371 patients for up to 12.3 years, showed that baseline sST2 levels of patients who died during follow up were higher than survivors, Meta-SMD of 0.502 [95% CI 0.273–0.730; p<0.0001, I^2^ = 88.78].

Soluble ST2 had a stronger association with risk of all-cause mortality in ACS (Four datasets, Meta-multivariate HR 2.207, 95% CI 1.160–4.198; p = 0.016, I^2^ = 95.661) than risk of all-cause mortality in HF (Fifteen datasets, Meta-multivariate HR 1.425, 95% CI 1.268–1.601; p<0.0001, I^2^ = 92.276).

Two acute ischaemic stroke studies also evaluated baseline sST2 levels in patients stratified by survival status. During 90 days follow up, 132 patients who died had higher baseline sST2 levels than the 903 who survived [Meta-SMD 1.151, 95% CI 0.670–1.633, p<0.0001, I^2^ = 83.474] [25, 26].

**Fig 6:**
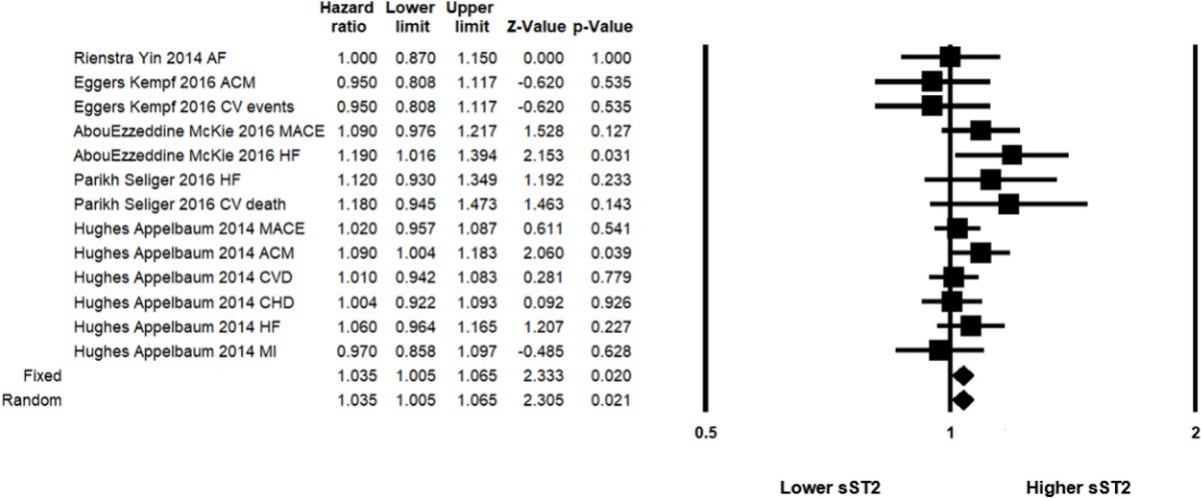
Summary forest plot showing the multivariate Meta-HR and 95% CI for risk of any adverse cardiovascular events in community populations and its relation to sST2 levels. The Meta-HR in the random effects model is shown by the black diamond at the bottom. The vertical line at 1 is the border for significance. AF = atrial fibrillation, ACM = all-cause mortality, CV = cardiovascular, HF = heart failure, CVD = cardiovascular disease, CHD = coronary heart disease, MI = myocardial infarction, MACE = major adverse cardiovascular events.

Five studies followed 18264 individuals from community cohorts for up to 15 years to evaluate risk of adverse events (including all-cause mortality, MACE, and occurrence of specific CVDs, such as development of AF) per log unit increase of sST2 levels (Fig 6). The Meta-multivariate HR was 1.035 [95% CI 1.005–1.065; p = 0.021, I^2^ = 2.114].

### Heterogeneity and meta-regression

Most of the studies had high heterogeneity (I^2^ value greater than 50%). For studies with high heterogeneity, meta-regression analyses (assessing the effect of age, follow up time, publication year and sST2 assay) did not identify covariates that reduced I^2^ value to below 50% in the vast majority of analyses.

## Discussion

We used systematic review methods to evaluate the associations between circulating levels of IL-33 and/or sST2 and CVD subtypes, and associations between these biomarkers and outcomes of mortality and MACE in CVD patients as well as community populations. Our main findings were that patients from the spectrum of CVDs have higher sST2 levels compared with healthy controls and that incremental increases in sST2 are associated with poor clinical outcomes of mortality and MACE over several years follow up in both CVD and community-based cohorts. We observed lower IL-33 levels in HF, CAD and ACS patients compared with controls. In contrast, higher IL-33 levels were recorded in acute ischaemic stroke patients compared with controls. There were insufficient data to examine the association of IL-33 with clinical outcomes in CVD or community populations.

This is the first meta-analysis to systematically evaluate both IL-33 and sST2 levels across the spectrum of CVDs. It suggests that peripheral circulating sST2 measurement may have clinical value in differentiating patients with CVD versus controls without CVD. The greatest difference in sST2 levels compared with controls was observed in AF and CHF.

Soluble ST2 is an FDA approved biomarker to evaluate prognosis of mortality in chronic heart failure. Previous meta-analyses of sST2 have also been performed in CAD and post-aortic valve replacement patients [14, 16, 18, 19] and showed that higher sST2 levels were associated with poor clinical outcomes in these populations. The current meta-analysis shows sST2 is associated with risk of poor outcomes of mortality, MACE and adverse cardiovascular events in a wide definition of CVD patients as well as community populations without identified cardiovascular disease. It suggests sST2 may also have value as a risk biomarker in CVD patients generally (besides chronic heart failure) and in community cohorts, although notably to a lesser extent than in CVD. Despite methodological differences between a meta-analysis by Liu *et al*. of ST2 (in ACS and CAD patients only) and our current study, meta-multivariate HRs of all-cause mortality in ACS patients are similar, with a respective meta-multivariate HR of 2.48, (95% CI 1.99–2.97) for Liu *et al*. and HR 2.207, (95% CI 1.160–4.198) in this current study [19].

To the best of our knowledge, this is the first meta-analysis of CVD studies of IL-33 and included 1638 participants. It suggests potential clinical value in measurement of IL-33, in that IL-33 levels were lower in CAD, HF and ACS patients versus controls. However, it must be noted other individual studies (not included in this meta-analysis after considering inclusion/exclusion criteria) have shown different results, with IL-33 levels being higher in heart failure patients compared with controls [27, 28]. Interestingly, IL-33 levels were higher in stroke patients compared with controls; in contrast to findings from other CVD subtypes, based on five studies with a sample size of 908. The reasons for these contrasting results in stroke are currently unclear and require further investigation. The low sample size of such studies indicate it would be premature to suggest a different role for IL-33 in cerebrovascular disease compared to other cardiovascular diseases. Moreover, a major drawback to advancing understanding of IL-33 in human cardiovascular health and disease is that often levels are below the detection limit of an assay and there is wide divergence in the methodology of how studies processed and analysed IL-33 samples in the current published literature [20]. In this meta-analysis, several IL-33 studies were not included due to levels of IL-33 being below the detection limit of the assay [24, 29, 30]. A further point of consideration is that IL-33 exists extracellularly in both full length and cleaved forms and cleavage may either enhance its potency or inactivate it depending on whether serine proteases or apoptotic caspases facilitate cleavage. In this meta-analysis, the included studies did not identify whether full length or cleaved forms of IL-33 were measured.

This meta-analysis showed that circulating IL-33 levels were much lower than sST2 levels, which reflects sST2’s role as a decoy receptor to limit IL-33 activity. Moreover, IL-33 exerts its effects in an autocrine/paracrine manner, and is quickly oxidised after its release from cells, making it more difficult to detect in circulation [31]. Due to these factors, sST2 is likely a much more clinically useful and attainable biomarker to measure in clinical studies as shown by the results of this meta-analysis. Overall, it remains to be elucidated whether IL-33 itself is cardioprotective or deleterious as suggested by contrasting pre-clinical animal studies. There is a need to advance understanding of the IL-33/ST2 axis in CVDs, and studying local tissue expression of IL-33 and the effects of blocking IL-33 or ST2 may be helpful [32, 33]. Of interest, a loss of function in the IL-33 gene has been shown to be protective against asthma in humans, with no harmful effects (i.e. cardiovascular abnormalities) reported [34].

This study has several limitations. The first is the low number of IL-33 clinical studies available which limits conclusions we can draw regarding IL-33’s role in cardiovascular health. Additionally, differences in the sensitivity of IL-33 assays are a potential hindrance for repeatability and interpretation of clinical studies. The second is that Meta-multivariate HRs reported are adjusted for different factors at the individual study level that could impact IL-33/sST2 expression (e.g. age, gender, comorbidities, body mass index, and levels of other inflammatory biomarkers). The majority of studies adjusted for age and gender; and many included extensive adjustment for other cardiovascular risk factors. Furthermore, the length of storage time for biomarkers before analysis varied, which may impact biomarker stability.

## Conclusions

In summary, this meta-analysis is the first to analyse the role of both IL-33 and sST2 across the spectrum of human CVDs. We showed that sST2 has diagnostic and prognostic value over several years of follow up across the spectrum of CVDs. Similarly, IL-33 shows some promise as a biomarker to differentiate between CVD patients and controls. Meta-SMDs showed that IL-33 levels are lower in HF and CAD patients compared to controls, while the reverse was observed in stroke patients. This observation and the small sample size means that further clinical studies are needed to determine if measurement of circulating IL-33 has diagnostic or prognostic value (similar to that of sST2) across the spectrum of CVD subtypes.

## Supporting information

S1 File Inclusion/exclusion criteria. (PDF)

S2 File QUADAS-2 scoring criteria. (PDF)

S3 File Table of studies. (PDF)

S1 Checklist PRISMA 2009 checklist. (PDF)
