# Supplementary material for: Role of the IL-33/ST2 axis in cardiovascular disease: A systematic review and meta-analysis
Source: PLoS One. 2021 Nov 1;16(11):e0259026. doi: 10.1371/journal.pone.0259026 (PMC8559957; doi:10.1371/journal.pone.0259026)
Supplement: S1 File — (PDF) [file pone.0259026.s001.pdf]

Primary screening (title and abstract)

Article topic must directly examine IL-33 and/or ST2 and its relationship to cardiovascular disease (exclude vascular disease localised to renal or hepatic system, exclude vasculitis, exclude autoimmune disorders, exclude transplant related diseases, exclude parasite diseases, exclude cancer, exclude HIV, exclude MRI studies, exclude obesity studies, exclude exercise studies)

Paper must be English language and have full text available (University of Cambridge access)

Must not be a comment or panel discussion or conference abstract

Secondary screening (full text)

Inclusion criteria

Completed clinical studies looking at protein levels of IL-33 and/or ST2 in one of plasma/serum/blood in patients (any age, gender, and ethnicity) with cardiovascular disease and/or healthy control populations.

For meta-analysis risk of event studies, HRs are the only accepted outcome measure and must be based on continuous and log transformed biomarker levels

Studies must be at least one of: Patient/control comparison, event/no event comparison, risk of event during follow up with HR as outcome measure

Biomarker levels at single time-points must be available

Post-hoc analyses are accepted

Exclusion criteria

Qualitative studies

Papers published before 2000

Genetic studies

Review papers

Animal studies

No HRs based on quadratic transformed data

No ORs or RRs for meta-analysis risk of event studies

No cardiomyopathy, paediatric, congenital disease, valvular disease studies, pulmonary hypertension studies

Non-extractable raw data (graphical depiction of data only, biomarker levels below detection limits of assay, unable to calculate biomarker mean and SD from data)

Biomarker levels collected from patients immediately after major surgery

Example search strategy: Pubmed

The authors carried out independent searches in Pubmed (including MEDLINE) in September 2020. The search terms were one of IL-33/Interleukin-33/ST2 combined with one of cardiovascular disease, stroke, myocardial infarction, heart failure, coronary disease, ischaemic heart disease and hypertension. The limits were set to human studies only, no publications before 2000, no reviews.

The inclusion/exclusion criteria were then applied to the search results.
