# Supplementary material for: Role of the IL-33/ST2 axis in cardiovascular disease: A systematic review and meta-analysis
Source: PLoS One. 2021 Nov 1;16(11):e0259026. doi: 10.1371/journal.pone.0259026 (PMC8559957; doi:10.1371/journal.pone.0259026)
Supplement: S2 File — (PDF) [file pone.0259026.s002.pdf]

QUADAS-2

Phase 1: State the review question: Is circulating IL-33 and ST2 associated with CVD and does it predict CV outcomes

		Quality assessment score
1.	Study design (cohort or case controlled)	/1
2.	Quality of inclusion criteria for selecting representative population *and* clear disclosure of inclusion criteria	/2
3.	Quality of exclusion criteria for excluding non-representative participants *and* disclosure of exclusion criteria listed	/2
4.	Quality points for cross-sectional matched control defined for datasets that reported patient and control samples. 2 characteristics=2<br><br>**OR**<br><br>Quality points for cohort study of population representing based on demographic characteristics (age, gender, BMI, disease definition, comorbidities)	/2
5	CVD clearly defined. Points assigned for how defined (diagnostic result, clinical code) and transparency in data collection and reporting	/2
6	Method for measurement of biomarker *and* analysis method for biomarker level fully disclosed (i.e log, mean/median SD/range values)	/2
7	Quality points for time points of study: cross-sectional time-point of test identical for all participants (1), related to index test if appropriate and reported (1).<br><br>**OR**<br>Time point for longitudinal study, >=12 months FU=2, 6-12 months=1, <6 months =0	/2

8	Representative sample. Points assigned for transparency of sample selection methods and applicability of the population representing. E.g. if heart failure, only those with severe (NHYA III-IV) so not representative of all heart failure patients so would lose a point.	/2
	**Total**	/15
