# Supplementary material for: Role of the IL-33/ST2 axis in cardiovascular disease: A systematic review and meta-analysis
Source: PLoS One. 2021 Nov 1;16(11):e0259026. doi: 10.1371/journal.pone.0259026 (PMC8559957; doi:10.1371/journal.pone.0259026)
Supplement: S3 File — (PDF) [file pone.0259026.s003.pdf]

## S3 Table

| Author-Date | Country of study population | Inclusion criteria | Exclusion criteria | Follow up and HRs (95% CI) | Primary outcome | Secondary outcome | Sample size | Mean age of patients (years) | Study type | Biomarker measured | Mean biomarker levels in patients (pg/ml) | Mean biomarker levels in controls (pg/ml) | QUADAS score |
|---|---|---|---|---|---|---|---|---|---|---|---|---|---|
| (Liu, Shen et al. 2013) | China | Coronary artery disease | No patients with history of cardiomyopathy, valvular heart disease, tumours, inflammatory diseases, liver/renal dysfunction, cerebral vessel diseases, peripheral angiopathy, pregnancy | None | Levels of IL-33 and IL-6 | None | 113 | Overall: 56.5±12.54 | Case control | Serum IL-33<br><br>ELISA Nanjing Jiancheng Technology Industry<br><br>5 ng/L sensitivity | Blood taken at admission<br><br>ACS: 78.60±44.84<br><br>Stable angina: 102.58±37.21 | 130.24±10.17 | 15 |
| (Liu, Shen et al. 2014) | China | Coronary heart disease | No patients with history of cardiomyopathy, valvular heart disease, severe renal disease, respiratory insufficiency, severe hepatic disease, malignancy, infection, cerebral vessel diseases | None | Levels of IL-33 and MMP-28 | None | 143 | AMI: 58.89±12.74<br><br>Unstable angina pectoris: 56.41±7.5<br><br>Stable angina pectoris: 55.24±8.39<br><br>Excluding stable, overall mean is 57.53±10.18 | Case control | Serum IL-33<br><br>ELISA Nanjing Jiancheng Technology Industry<br><br>5 ng/ml sensitivity | Blood taken at admission<br><br>AMI: 71.38±6.63<br><br>Unstable angina pectoris: 83.13±17.99<br><br>Stable angina pectoris: 102.58±37.21<br><br>Overall IL-33 is 88.17±29.21<br><br>Excluding stable, overall mean is 77.84±15.15 | 130.24±10.17 | 15 |
| (Guzel, Serin et al. 2013) | Turkey | NSTEMI | No patients with renal failure, collagen tissue disease, infection, vasculitis, depression, undergone angioplasty, bypass surgery, open heart surgery | None | Levels of IL-33 and other markers | None | 80 | 52.36±9.4 | Case control | Serum IL-33<br><br>ELISA USCN Life Science, Houston<br><br>6.4 pg/ml sensitivity | Blood collected at admission and 12, 24, 48H after percutaneous coronary intervention<br><br>At admission, 94.46±32.7<br><br>At 12H: 126.82±44.5<br><br>At 24H: 119.55±32.73<br><br>At 48H: 121.74±41.38<br><br>25 patients had unstable angina: At admission, 115.36±37.16<br><br>Combined unstable angina and NSTEMI is 103.96±36.03 | At admission, 122.81±47.16 | 15 |
| (Zhang, Fan et al. 2020) | China | Patients undergoing coronary angiography | No patients with history of severe HF, acute infections, autoimmune diseases, malignancy, thyroid dysfunction, severe | None | Levels of ST2 | Logistic regression analysis showing ST2 is | 262 | ACS: 66.32±12.03 | Case control | Serum ST2<br><br>ELISA R&D systems | Baseline<br><br>ACS: 21657±12786 | 16229±6564 | 15 |

| | | | | | | | | | | | | | |
|---|---|---|---|---|---|---|---|---|---|---|---|---|---|
| | | Performed for suspected CAD<br><br>97 had acute coronary syndrome (ACS), 63 had stable angina | hepatic/renal insufficiency, use of lipid lowering drugs within 3 months | | | independently diagnostic | | Stable angina: 65.65±10.08<br><br>Total patients (N=262): 64.18±11.19 | | | Stable angina: 17330±7232<br><br>Total ST2 level is 19953.24±11117.46 | | |
| (Demyanets, Speidl et al. 2014) | Austria | Coronary artery disease | No patients with autoimmune disease, chronic infections, hepatic/renal disorder | Mean follow up of 43 months | Levels of IL-33 and ST2 | Mortality during follow up | 438 | Stable angina: 65.9±11.5<br><br>NSTEMI: 64.6±13.5<br><br>STEMI: 62.9±13.7 | Case control | Serum ST2<br><br>ELISA R&D systems<br><br>31.3 pg/ml sensitivity | Blood taken directly before coronary angiography<br><br>Stable angina: Median is 169<br>Mean calculated to be 169.33±135.28<br><br>NSTEMI: Median is 269<br>Mean calculated to be 307.33±255.11<br><br>STEMI: Median is 453<br>Mean calculated to be 484.67±282.16<br><br>Overall ST2 is 288.07±251.57<br><br>37 dead: Median is 415<br>Mean calculated to be 569.67±788.19<br><br>336 survivors: Median is 241<br>Mean calculated to be 265.33±221.12<br><br>Excluding stable angina, overall is 396.45±282.69 | Median is 163<br><br>Mean calculated to be 179±110.69 | 14 |
| (Barbarash, Gruzdeva et al. 2016) | Russia | Myocardial infarction | No patients with diabetes, anaemia, renal/hepatic failure, acute infectious/inflammation diseases, autoimmune disease, long term corticosteroid therapy, death during hospitalisation | 12 day observation for unfavourable outcomes during hospital stay | Levels of ST2 and other biomarkers | Occurrence of angina, rhythm disturbance, acute HF during hospital stay | 118 | Median is 58<br>Mean age calculated to be 59±6.78 | Case control | Serum ST2<br><br>Presage ST2 | Blood taken at admission<br><br>Median on Day 1 was 44750<br>Mean calculated to be 54403.33±51749.51<br><br>Median on Day 12 was 17820<br>Mean calculated to be 18790±5991.97<br><br>For favourable outcomes (58 patients), median on Day 1 was 35450, Day 12 was 17000<br>Mean calculated to be 37893.33±22312.54 | Median of 18810<br>Mean calculated to be 18320±4600.46 | 13 |

| | | | | | | | | | | | | | |
|---|---|---|---|---|---|---|---|---|---|---|---|---|---|
| | | | | | | | | | | | on Day 1, 17540±4606.95 on Day 12<br><br>For unfavourable outcomes (30 patients), median on Day 1 was 69990, Day 12 was 20200 Mean calculated to be 110686.67±132588.32 on Day 1, 25483.33±18144.98 | | |
| (Wang, Tan et al. 2018) | China | Acute myocardial infarction and acute aortic dissection | No patients with aortic trauma, pseudo aneurysm, history of heart failure, renal dysfunction, severe pulmonary diseases, cancer. Also no patients who received packed red blood cells, whole blood or platelets less than 10 days before blood sample is taken. | None | Levels of ST2 in acute aortic dissection and myocardial infarction patients | None | 301 | 53.09±11.63 | Case control | Serum ST2<br><br>DuoSet ELISA R&D systems<br><br>0.019 ng/ml sensitivity | Baseline<br><br>Median is 14660 Mean calculated to be 15943.33±10047.78 | Median is 6720 Mean calculated to be 6836.67±2719.95 | 12 |
| (Pfetsch, Sanin et al. 2017) | Germany<br><br>Part of KAROLA study cohort | Patients who were admitted within 3 months of an acute coronary event or CABG and are now stable | None | Median of 12.3 year follow up<br><br>Multi-MACE HR per 1 SD log sST2: 1.01 (0.89-1.15)<br><br>Adjustments: Age, gender, school education, rehabilitation clinic, smoking status, history of diabetes mellitus, PCI, left ventricular function, HDLcholesterol, LDL-cholesterol, eGFR (cystatin C based), NT-pro-BNP (log transformed), hs-CRP (log transformed), hs-TnI (log transformed) | Levels of ST2 | Fatal and non-fatal cardiovascular events, total mortality and recurrent cardiovascular events during follow up | 1081 | 58.9±8 | Observational | Serum ST2<br><br>ELISA ST2 Critical Diagnostics | Blood taken at baseline during discharge from rehabilitation (average of 43 days after acute event)<br><br>Median is 28980 Mean calculated to be 29306.67±8388<br><br>821 survivors: Median is 28580 Mean calculated to be 28746.67±7797.5<br><br>260 dead: Median is 31460 Mean calculated to be 31463.33±10011.69 | | 12 |
| (Kohli, Bonaca et al. 2012) | 17 countries, part of MERLIN-TIMI 36 trial (studied Ranolazine) | Non-ST elevation acute coronary syndrome | No patients with end stage renal disease, significant hepatic disease, cardiogenic shock | 1 year follow up<br><br>Multi-HR all-cause mortality per log sST2: 1.26 (1.12-1.4)<br><br>Multi-HR HF risk per log sST2: 1.37 (1.21-1.55)<br><br>Adjustments: TIMI risk score covariates, as defined by age >65 years, known coronary artery disease, diabetes, hypertension, dyslipidemia, severe angina (⩾2 episodes in 24h), ST changes | Levels of ST2 | Cardiovascular disease, heart failure, all-cause mortality, during follow up | 4426 | Unstated | Clinical trial | Serum ST2<br><br>Presage ST2 | Baseline<br><br>Median is 24400 Mean calculated to be 117362.5±60469.24 | | 12 |

| | | | | | | | | | | | | | |
|---|---|---|---|---|---|---|---|---|---|---|---|---|---|
| | | | | ⩾0.5 mm, and aspirin use<br><br>Uni-HR all-cause mortality per log sST2: 1.56 (1.41-1.73) | | | | | | | | | |
| (Dieplinger, Egger et al. 2014) | Germany, part of LURIC cohort | Coronary artery disease (stable) | No patients with acute illness other than acute coronary syndrome, malignancy within 5 years | Median of 9.8 years follow up | Levels of ST2 | All-cause mortality and cardiovascular mortality during follow up | 1345 | Median is 65<br>Mean calculated to be 64.67±9.65 | Observational, post-hoc | Serum ST2<br><br>Presage ST2 | Blood taken before angiography<br><br>Median is 19400<br>Mean calculated to be 19900±6604.74<br><br>477 dead: Median is 21400<br>Mean calculated to be 22100±8699.78<br><br>868 survivors: Median is 18500<br>Mean calculated to be 19033.33±5791.87 | | 14 |
| (Jacobs, Alam et al. 2018) | USA | CAD (patients presenting for CABG), 55% was unstable angina | No patients who were not discharged alive | 1 year follow up<br><br>Multi-HR MACE per log sST2: 1.14 (0.95-1.36)<br><br>Adjustments: The Society of Thoracic Surgeons 30-day readmission model and The Society of Thoracic Surgeons ASCERT long-term mortality risk model | Occurrence of MACE during follow up | Levels of ST2 | 1047 | Readmitted or dead: 63.91±9.92<br><br>Not readmitted or dead: 67.8±9.98<br><br>Combined mean: 65.41±10.11 | Observational | Serum ST2<br><br>Custom made multiplex ELISAs (Meso Scale Discovery) | Blood taken before CABG<br><br>Median is 3800<br>Mean calculated to be 14912.5±8082.72 | | 10 |
| (Lepojarvi, Huikuri et al 2018) | Lepojarvi et al based in Finland, part of ARTEMIS trial (studied diabetes and cardiovascular issues). | Stable coronary artery disease | No patients with NYHA 4, significant valvular disease, end stage renal failure. Also no patients with permanent pacemaker, life expectancy less than 1 year, younger than 18, older than 85. | Mean follow up of 76±20 months | Levels of ST2 and other biomarkers | All-cause mortality and sudden cardiac death during follow up | 1945 | Survivors: 66±9<br><br>Deaths: 72±8 | Observational | Serum ST2<br><br>ELISA Quantikine R&D systems | Survivors: Median is 16300<br>Mean calculated to be 16866.67±6454.73<br><br>Deaths: 21500<br>Mean calculated to be 22066.67±10377.68 | | 13 |
| (Dhillon, Narayan et al. 2011) | UK | NSTEMI | No patients with malignancy, renal replacement therapy, surgery in the previous month. | Mean of 532 days follow up<br><br>Multi-HR all-cause mortality per $log_{10}$ sST2: 2.03 (1.01-4.09)<br><br>Multi-HR HF risk per per $log_{10}$ sST2: 1.64 (0.68-3.95)<br><br>Adjustments: Age, gender, previous angina or acute myocardial infarction, HF, hypertension, diabetes, Killip class, eGFR, log10 glucose level, therapy with βblockers, or statins, | Levels of IL-33 and ST2 | MACE, death, heart failure and myocardial infarction during follow up | 577 | 70±13 | Observational | Serum IL-33 and ST2<br><br>R&D systems, Abingdon | Blood collected at 3-5 days after admission<br><br>Median ST2 is 638 (range of 82-18875)<br>Mean calculated to be 5058.25±3064.19<br><br>304 patients (52.7%) had very low IL-33, below the 5.4 detection limit of the assay.<br>In the remaining 273 patients (47.3%), levels ranged from 5.8-8966.7 | | 10 |

| | | | | | | | | | | | | | |
|---|---|---|---|---|---|---|---|---|---|---|---|---|---|
| | | | | log10 troponin I, previous history of hypercholesterolemia, or smoking<br><br>Uni-HR all-cause mortality per $log_{10}$ sST2: 4.76 (2.61-8.66) | | | | | | | | | |
| (Dhillon, Narayan et al. 2013) | UK | STEMI | No patients with malignancy, renal replacement therapy, surgery in previous month. | Median follow up of 587 days<br><br>Multi-HR all-cause mortality per $log_{10}$ sST2: 3.15 (1.56-6.36)<br><br>Multi-HR HF risk per $log_{10}$ sST2: 2.05 (0.96-4.38)<br><br>Adjustments: Age, gender, previous history of angina/AMI, hypertension, diabetes, Killip Class, eGFR, peak creatinine phosphokinase level, treatment with thrombolysis, betablockers, statins, ACE inhibitors or ARBs<br><br>Uni-HR all-cause mortality per $log_{10}$ sST2: 9.07 (5.15-15.99) | Levels of IL-33 and ST2 | All-cause mortality, heart failure and recurrent infarction during follow up | 677 | 64±12.2 | Observational | Serum IL-33 and ST2<br><br>R&D systems<br><br>2.2 pg/ml sensitivity for ST2<br><br>5.4 pg/ml sensitivity for IL-33 | Blood collected 3-5 days after admission<br><br>IL-33 levels were very low in 364 patients (53.8%). These had IL-33 levels below detection limit of 5.4. The remaining 313 (46.2%) had IL-33 ranging from 6.3-17893.7<br><br>Median ST2 was 677, mean calculated to be 4677.25±2767.66<br><br>For the patients that died, median ST2 was 1125, mean calculated to be 4538.5±3404.20<br>For the patients that survived, median ST2 was 630, mean calculated to be 3262±1889.89 | | 10 |
| (Zagidullin, Motloch et al. 2020) | Russia | STEMI | No patients presenting later than 48H of symptoms, severe valvular dysfunction, stenosis in valves, atrial flutter, permanent atrial fibrillation, pacemaker, malignancy, acute pulmonary embolism, severe COPD, uncontrolled bronchial asthma, acute infection, pneumonia, kidney failure, acute bronchitis, pregnancy. | 2 year follow up | Levels of ST2 and other biomarkers | Cardiovascular mortality during follow up | 147 | 60.9±12.1 | Observational | Serum ST2<br><br>Biomedica Slovakia | Blood collected at admission<br><br>Median is 43800<br>Mean calculated to be 41700±23733.72<br><br>In the 33 patients that died (22.4%), ST2 was 93700±97100<br><br>In the 114 patients that survived (77.6%), ST2 was 51300±47300 | | 15 |
| (Shimpo, Morrow et al. 2004) | Part of TIMI and STEMI (ENTIRE)-TIMI 23 cohorts | Acute myocardial infarction | No patients with risk of haemorrhage, severe renal insufficiency and cardiogenic shock | 30 day follow up for mortality | Levels of ST2 | Mortality, recurrent MI, heart failure during follow up | 810 | Quartiles combined: Mean calculated to be 58±10.24 | Observational | Serum ST2<br><br>ELISA Medical and Biological Laboratories | Blood taken within 24H of enrolment<br><br>Dead: Median is 379<br>Mean calculated to be 419±268.72<br><br>Alive: Median is 233<br>Mean calculated to be 250.33±120.31 | | 12 |
| (Zhang, Zhang et al. 2013) | China | Acute myocardial infarction | None | 6 month follow up | Levels of IL-33 and ST2 | Recurrence of MI or mortality | 95 | Data not available | Case control | Serum IL-33 and ST2<br><br>R&D ELISA | Blood collected within 24H of acute attack | ST2 levels read off the graph: 12000 | 8 |

| | | | | | | | | | | | | | |
|---|---|---|---|---|---|---|---|---|---|---|---|---|---|
| | | | | | | during follow up | | | | | ST2 levels read off the graph: 35000<br><br>IL-33 levels read off the graph: 450<br><br>For 25 patients, negative event occurred, their median ST2 level was 42540, mean calculated to be 48630±48778.63<br>Median IL-33 level was 405.24, mean calculated to be 581.38±517.49<br><br>For 34 patients, no negative event occurred, their median ST2 level was 18010, mean calculated to be 25676.67±37443.03<br>Median IL-33 level was 381.99, mean calculated to be 739.16±1019.30 | IL-33 levels read off the graph: 440 | |
| (Gerber, Weston et al. 2017) | USA | Myocardial infarction | Not stated | 1 year follow up<br><br>Multi-HR all-cause mortality per log sST2: 3.17 (2.61-3.85)<br><br>Adjustments: Age, sex<br><br><br>Uni-HR all-cause mortality per log sST2: 3.59 (3-4.31) | Mortality during follow up | Levels of ST2 | 1401 | 64.6±13.8 | Observational | Serum ST2<br><br>Presage | Blood taken at index MI<br><br>Median is 49000 | | 11 |
| (Wang, Wang et al. 2017) | China | Acute myocardial infarction | No patients with other heart disease, infection, abnormal pulmonary artery or aorta, liver/kidney dysfunction, malignancy | 1 year follow up | Levels of IL-33 and ST2 | MACE during follow up | 180 | MACE: 61.93±9.29<br><br>No MACE: 61.15±8.74<br><br>Overall mean: 61.41±8.9 | Observational | Serum IL-33 and ST2<br><br>ELISA Abbott Laboratories USA | Blood taken at admission<br><br>MACE: IL-33 is 433.27±95.64<br><br>ST2 is 823.47±149.25<br><br>No MACE: IL-33 is 372.25±84.25<br><br>ST2 is 601.83±125.84 | | 12 |
| (Yu, Oh et al. 2017) | South Korea | STEMI treated by PCI | No patients with thrombolytic therapy, MI caused by stent thrombosis, allergy to antiplatelet, history of CAD, cardiomyopathy, chronic heart failure | 1 year follow up | Levels of ST2 and NT-proBNP | MACE during follow up | 323 | Overall: 59.1±13.1<br><br>MACE: 63.7±14.4<br><br>No MACE: 58.5±12.8 | Observational | Serum ST2<br><br>R&D systems | Blood taken at admission<br><br>Total: 75800±27800<br><br>MACE: 84000±29700<br><br>No MACE: 74700±27600 | | 12 |

| | | | | | | | | | | | | | |
|---|---|---|---|---|---|---|---|---|---|---|---|---|---|
| (Karimzadeh, Zamani et al. 2017) | Iran | Acute myocardial infarction (STEMI)<br><br>Increase in TnT levels and ST elevation in anterolateral and anteroseptal leads | No patients with chronic renal failure, autoimmune disease, cardiogenic shock | None | Levels of IL-33 and ST2 | Correlation of biomarker levels and ejection fraction | 81 | Not stated | Case control | Serum IL-33 BioLegend San Diego<br><br>Serum ST2 Abcam UK | Blood taken after inclusion/exclusion criteria considered<br><br>IL-33: 124.85±203.5<br><br>ST2: 419.83±285.22 | IL-33: 78±41.39<br><br>ST2: 248.01±181.27 | 13 |
| (Schernthaner, Lichtenauer et al. 2017)<br><br>Substudy (with ST2 levels analysed) of previous study: Recruitment of circulating dendritic cell precursors into the infracted myocardium and pro-inflammatory response in acute myocardial infarction | Germany | Myocardial infarction (STEMI and NSTEMI)<br><br>Angina pectoris symptoms and elevated serum creatine kinase and cardiac Troponin I | No patients with infections, malignancies, autoimmune diseases, hyperthyroidism, immunosuppression | Total cohort mean hospital stay of 5.5 days | Levels of ST2 | Correlation of biomarkers with other parameters | 194 | STEMI: 61.6±11<br><br>NSTEMI: 65.6±11.2<br><br>Combined: 63.53±11.23 | Case control | Serum ST2<br><br>DuoSet DY523 R&D systems | Blood taken after admission<br><br>STEMI (61 patients): Median is 13210.9 Mean calculated to be 37194.28±25108.68<br><br>NSTEMI (57 patients): Median is 11989.1 Mean calculated to be 33887.95±22345.13<br><br>Combined: 35597.15±23770.62 | Median is 5247.7 Mean calculated to be 6853.98±2827.10 | 13 |
| (Fhaid Mostafa 2019) | Egypt | Chronic heart failure | No patients with anaemia, fever, liver/renal dysfunction, diabetes, inflammatory diseases, autoimmune disease, cancer, pregnancy | 3 months follow up | Levels of ST2, IL-34, BNP, TNFα | Mortality during follow up | 60 | 55.97±8.27 | Case control | Serum ST2<br><br>ELISA RayBiotech Norcross USA | Blood taken at admission<br><br>419.95±49.03<br><br>After 12 weeks standard care: 298.37±84.5<br><br>Dead: 506.19±3.87<br><br>Survived: 419.95±49.03 | 100.19±9.9<br><br>After 12 weeks standard care: 99.33±11.11 | 13 |
| (Santhanakrishnan, Chong et al. 2012) | Singapore<br><br>SHOP cohort | Heart failure | No patients with HF primarily due to severe valve disease, primary diagnosis of acute coronary syndrome that caused transient acute pulmonary edema, end-stage renal failure, rare specific cause of HF e.g. congenital, isolated right HF, life threatening comorbidity | None | Levels of ST2 and other biomarkers | None | 151 | HFPEF: 69±12<br><br>HFREF: 59±11 | Case control | Serum ST2<br><br>Presage ST2<br><br>10.91 ng/ml sensitivity | HFPEF: Median is 31520 Mean calculated to be 36006.67±20914.55<br><br>HFREF: 35250 Mean calculated to be 39003.33±19437.81<br><br>Overall ST2 is 37519.83±20137.44 | Median is 27580 Mean calculated to be 27290±8617.71 | 15 |
| (Najjar, Faxen et al. 2019) | Sweden<br><br>From Karolinska Rennes study | Heart failure | No patients with cardiomyopathy, pericardial constriction, clinically significant pulmonary disease, end stage renal disease requiring dialysis, bi-ventricular pacemaker, anticipated cardiac surgery, anticipated percutaneous intervention on aortic stenosis | Median follow up of 204 days<br><br>Multi-HR MACE for HFpEF per log unit sST2: 6.62 (1.04-42.28)<br><br>Multi-HR MACE for HFrEF per log unit | Levels of ST2 | Mortality and MACE during follow up | 193 | Median is 63 Mean calculated to be 61±12.06 | Case control | Serum ST2<br><br>Presage ST2 | Blood taken at enrolment<br><br>HFrEF (86 patients): Median is 35000 Mean calculated to be 36666.67±21865.95<br><br>HFpEF (86 patients): Median is 23000, | Median is 25000 Mean calculated to be 26000±8745.37 | 11 |

| | | | | | | | | | | | | | |
|---|---|---|---|---|---|---|---|---|---|---|---|---|---|
| | | | | sST2: 3.51 (1.05-11.69)<br><br>Multi-HR all-cause mortality for HFpEF per log sST2: 7.32 (0.35-154.27)<br><br>Adjustments for HFpEF: Age, sex, and NYHA class<br><br>Uni-HR HFpEF all-cause mortality per log sST2: 12.39 (0.7-218.55)<br><br>Uni-HR HFpEF MACE per log sST2: 10.04 (1.89-53.44)<br><br>Uni-HR HFrEF MACE per log sST2: 3.28 (1.06-10.16) | | | | | | | mean calculated to be 23666.67±10555.98<br><br>Overall mean is 30166.67±18317.96 | | |
| (Aimo, Januzzi et al. 2020) | Merged multiple cohorts<br><br>Previous meta-analysis (Prognostic value of high-sensitivity troponin T in chronic heart failure: an individual patient data meta-analysis)<br><br>PEOPLE and SHOP cohorts | Heart failure | Not stated | 5 year follow up<br><br>Multi-HR all-cause mortality per ln sST2: 1.61 (1.33-1.95)<br><br>Multi-HR cardiovascular death per ln sST2: 1.51 (1.23-1.86)<br><br>Adjustments: Age, female sex, ethnic group, ischaemic heart failure, LVEF category (heart failure with reduced, mid-range, or preserved ejection fraction), New York Heart Association class I–II vs. III–IV, body mass index, estimated glomerular filtration rate, anaemia, hypertension, atrial fibrillation, diabetes, chronic obstructive pulmonary disease, therapy with beta-blockers, angiotensin-converting enzyme inhibitor/angiotensin receptor blockers, mineralocorticoid receptor antagonists | Levels of ST2 and other biomarkers | All-cause mortality, cardiac mortality and hospitalisation for heart failure during follow up | 5301 | Median is 66<br>Mean calculated to be 65.67±12.61 | Post-hoc | Serum ST2<br><br>Presage ST2 assay<br><br>1.3 ng/ml sensitivity | Median is 29<br>Mean calculated to be 31.33±15.57 | | 10 |
| (Lupon, de Antonio et al. 2013) | Spain | Heart failure | None | Median of 41.4 months follow up | Levels of ST2 and other biomarkers | All-cause mortality during follow up | 876 | Median is 70.3<br>Mean calculated to be 69.33±12.4 | Observational | Serum ST2<br><br>Presage ST2 | Blood taken at conventional hospital visits<br><br>Median is 38.1 | | 11 |

| | | | | | | | | | | | | | |
|---|---|---|---|---|---|---|---|---|---|---|---|---|---|
| | | | | | | | | | | | Mean calculated to be 39.93±14.92<br><br>311 dead: Median is 44.7<br>Mean calculated to be 46.6±20.03<br><br>565 alive: Median is 35.4<br>Mean calculated to be 36.73±12.04 | | |
| (Zhang, Zhang et al. 2015) | China | Heart failure (new presentation or decompensation of previously stable chronic HF) | No patients with acute coronary syndrome, cancer, acute pulmonary embolism | 1 year follow up<br><br>Multi-HR all-cause mortality per ln sST2: 2.09 (1.73-2.54)<br><br>Adjustments: Sex, age, diabetes mellitus, ischemic heart disease, prior heart failure, systolic blood pressure, New York Heart Association functional class, left ventricular ejection fraction, angiotensin-converting enzyme inhibitor or angiotensin II receptor blocker treatment, β-blockers treatment, hemoglobin and eGFR, NT-proBNP | Levels of ST2 and galectin-3 | All-cause mortality during follow up | 1161 | 58±15 | Observational | Serum ST2<br><br>Critical Diagnostics | Blood taken at admission<br><br>Median is 36900<br>Mean calculated to be 39033.33±22118.54 | | 13 |
| (Dupuy, Curinier et al. 2016) | France | Heart failure | No patients with unstable angina or acute coronary syndrome in last month, cardiac surgery, chemotherapy | Median follow up of 42.3 months<br><br>Uni-HR all-cause mortality per $\log_{10}$ sST2: 3.915 (2.31-6.635) | Levels of ST2 and CRP | All-cause mortality, cardiovascular death, HF during follow up | 178 | Median is 75.43<br>Mean calculated to be 74.34±11.06 | Observational | Serum ST2<br><br>Presage ST2 | Blood frozen and tested 4 years later<br><br>Median is 37400<br>Mean calculated to be 42200±37519.64<br><br>112 Alive: Median is 28220<br>Mean calculated to be 31740±25685.14<br><br>66 Dead: Median is 52400<br>Mean calculated to be 63300±57524.46 | | 13 |
| (Gul, Yucel et al. 2017) | Turkey | Heart failure | No patients with cancer, sepsis, ongoing systemic inflammatory conditions, pregnancy, autoimmune disease, glucocorticoid therapy, acute MI, cardiogenic shock | Mean follow up of 12±4 months | Occurrence of cardiovascular death during follow up | Levels of ST2 | 130 | 67±11 | Observational | ST2<br><br>Presage ST2<br><br>2 ng/ml lower detection limit | Assume blood taken at enrolment<br><br>Survivors (107 patients): Median is 27000<br>Mean calculated to be 65250±37464<br><br>Deceased (23 patients): Median is 51000<br>Mean calculated to be 71250±36552.41 | | 13 |

| | | | | | | | | | | | | | |
|---|---|---|---|---|---|---|---|---|---|---|---|---|---|
| (Segiet, Romuk et al. 2019) | Poland | Heart failure (with reduced ejection fraction)<br><br>Includes stable and unstable (worsened symptoms 1 month before inclusion) heart failure | No patients with autoimmune disease, chronic or acute infection, recent immunosuppression, MI within 3 months, congenital heart disease, end stage renal failure, advanced liver cirrhosis, hyperthyroidism, acute respiratory distress syndrome, cancer, pregnancy | None | Levels of IL-33 | None | 215 | 65.39±12.75 | Case control | Serum IL-33<br><br>ELISA LEGEND MAX BioLegend<br><br>4.14 pg/ml sensitivity | Blood taken at admission<br><br>Median is 16.91<br>Mean calculated to be 32.64±60.61 | Median is 92.51<br>Mean calculated to be 188.58±308.39 | 14 |
| (Firouzabadi, Dashti et al. 2020) | Iran | Heart failure (HFrEF) | No patients with severe valvular heart disease, GFR <30 mL/min or significant comorbidities | None | Levels of IL-33 and ST2 | Differences in biomarker levels in patients on beta-blockers and carvedilol | 66 | 66±11.1 | Case control | Serum IL-33<br><br>Ab119547-IL-33 ELISA Abcam company<br><br>0.2 pg/ml sensitivity<br><br>Serum ST2<br><br>ST2 ELISA Elabscience Company<br><br>0.19 ng/ml sensitivity | Assume blood taken at enrolment<br><br>No units given for biomarkers<br><br>IL-33: 2.45±0.69<br><br>ST2: 3416.6±1089.1 | No units given for biomarkers<br><br>IL-33: 2.67±0.33<br><br>ST2: 2971.6±792.5 | 12 |
| (Bahuleyan, Alummoottil et al. 2018) | India | Heart failure (HFrEF)<br><br>Had combination of chronic and acute heart failure | No patients with recent acute coronary syndrome, coronary revascularisation within last 2 months, myocarditis, cardiogenic shock, advanced liver/renal disease, malignancy | 1 year follow up<br><br>Multi-HR MACE per ln sST2: 2.046 (1.246-3.358)<br><br>Adjustments: Age, body mass index, diabetes, dyslipidemia, creatinine clearance, smoking, NYHA functional class, use of angiotensin converting enzyme inhibitors (ACEI) or angiotensin receptor blockers (ARB), b-blockers | Levels of ST2 | Cardiovascular death, hospitalisation for HF during follow up | 141 | 60.3±10.4 | Observational | Serum ST2<br><br>Presage ST2 | Blood collected at enrolment<br><br>71700±83900<br><br>57 Adverse outcome: 106600±116200<br><br>84 Non-adverse outcome: 48000±36800 | | 12 |
| (Zhang, Zhang et al. 2014) | China | Heart failure (de novo presentation or worsening of previous chronic HF) | No patients with acute coronary syndrome, cancer, acute pulmonary embolism | Median of 19.1 months follow up<br><br>Multi-HR MACE per ln sST2: 1.52 (1.3-1.78)<br><br>Adjustments: Age, systolic blood pressure, body mass index, NYHA functional class, left ventricular ejection fraction, sodium, total cholesterol, blood urea nitrogen, total bilirubin, albumin, NT-proBNP and loop diuretics treatment | Levels of ST2 | All-cause mortality and cardiac transplantation during follow up | 1528 | 58±16 | Observational | Serum ST2<br><br>Critical Diagnostics | Blood taken at admission<br><br>Median is 37100<br>Mean calculated to be 39333.33±22631.27<br><br>325 adverse events: Median is 55600<br>Mean calculated to be 65200±50188.44<br><br>1203 no adverse events: Median is 33600 | | 13 |

| | | | | | | | | | | | | | |
|---|---|---|---|---|---|---|---|---|---|---|---|---|---|
| | | | | Uni-HR MACE per ln sST2: 2.73 (2.42-3.07) | | | | | | | Mean calculated to be 34966.67±17293.26 | | |
| (Xu, Su et al. 2014) | China | Heart failure (NYHA 2-4) | No patients with MI within 1 month, moderate/severe valvular disease, congenital heart disease, chronic inflammatory disease, thyroid disease, malignancy | 1 year follow up<br><br>Multi-HR MACE per $log_2$ sST2: 2.19 (1.55-3.1)<br><br>Adjustments: Age, heart rate, BMI, creatinine, NT-pro BNP, NYHA class (II versus III/IV), and history of hypertension | Levels of ST2 | All-cause mortality and hospitalisation for HF during follow up | 233 | 62±11 | Observational | Serum ST2<br><br>Critical Diagnostics | Baseline<br><br>Median is 35600<br>Mean calculated to be 37500±14993.74 | | 12 |
| (Bayes-Genis, Pascual-Figal et al. 2010) | Spain | Heart failure (recently destabilised) | No patients with acute coronary syndrome within 3 months, pending cardiac transplant or revascularisation, severe valvular disease, end stage pulmonary/hepatic/renal disease | 1 year follow up | Levels of ST2 | All-cause mortality, heart failure hospitalisation, cardiac transplantation during follow up | 48 | No event: 62±12<br><br>Event: 62±15 | Observational | Serum ST2<br><br>ELISA Medical and Biological Laboratories, Woburn | Baseline<br><br>Median is 270<br>Mean calculated to be 300±236.91<br><br>After 2 weeks, median is 230<br>Mean calculated to be 213.33±145.21<br><br>No event (22 patients): Baseline median is 310, mean calculated to be 410±396.24<br><br>Event (26 patients): Baseline median is 230, mean calculated to be 266.67±243.14 | | 12 |
| (Moliner, Lupon et al. 2018) | Multicentre, from well-established cohort also used in the below studies<br><br>Combined use of high sensitivity ST2 and NTproBNP to improve the prediction of death in heart failure<br><br>Soluble neprilysin is predictive of cardiovascular death and heart failure hospitalisation in heart failure patients<br><br>Combined use of the novel biomarkers high sensitivity troponin T and | Chronic heart failure | Not stated | Mean follow up of 4.9±2.8 years<br><br>Uni-HR MACE HFrEF per 1 SD log sST2: 1.45 (1.31-1.61)<br><br>Uni-HR MACE HFmEF per 1 SD log sST2: 1.77 (1.32-2.36)<br><br>Uni-HR MACE HFpEF per 1 SD log sST2: 1.11 (0.86-1.43) | Levels of ST2 and other biomarkers | All-cause mortality, cardiovascular mortality, MACE during follow up | 1069 | HFrEF: 65.5±12.6<br><br>HFmrEF: 67±12<br><br>HFpEF: 69.6±14.4 | Observational | Serum ST2<br><br>Presage ST2 | Blood collected at conventional ambulatory visits<br><br>HFrEF: Median is 37500<br>Mean calculated to be 39100±14110.42<br>HFmrEF: Median is 37100<br>Mean calculated to be 38333.33±14763.57<br>HFpEF: 44400<br>Mean calculated to be 44666.67±18733.99<br><br>Overall mean calculated to be 39706.89±14958.45 | | 10 |

| | | | | | | | | | | | | | |
|---|---|---|---|---|---|---|---|---|---|---|---|---|---|
| | ST2 for heart failure risk stratification vs conventional assessment | | | | | | | | | | | | |
| (Broch, Ueland et al. 2012) | Multicentre, 21 countries<br><br>Substudy of CORONA study, which investigated the effect of Rosuvastatin in systolic HF | Heart failure (patients must be over 60 years old) | No patients with recent cardiovascular events, current/planned operations, liver disease, alanine transamine more than 2 times greater than upper limit of normal, serum creatine ≥2.5 mg/dL, chronic muscle disease, contraindication to statins, unexplained creatine kinase ≥2.5 upper limit of normal, thyroid stimulating hormone ≥2 upper limit of normal, any condition substantially reducing life expectancy | Median follow up of 2.6 years<br><br>Multi-HR MACE per ln sST2: 1.16 (0.93-1.44)<br><br>Multi-HR all-cause mortality per ln sST2: 1.12 (0.91-1.38)<br><br>Multi-HR cardiovascular death per ln sST2: 1.12 (0.89-1.41)<br><br>Adjustments: Age, gender, diabetes mellitus, a history of intermittent claudication, body mass index, heart rate, left ventricular ejection fraction, and New York Heart Association class, as well as baseline estimated glomerular filtration rate and the ratio of apolipoprotein (Apo) A1 to ApoB, baseline N-terminal pro brain natriuretic peptide and C-reactive protein<br><br>Uni-HR all-cause mortality per ln sST2: 2.03 (1.72-2.4)<br><br>Uni-HR MACE per ln sST2: 1.99 (1.68-2.36) | Occurrence of MACE, CV death, all-cause mortality during follow up | Levels of ST2 | 1449 | 72±7 | Observational | Serum ST2<br><br>Presage | Blood taken at baseline<br><br>Median is 17800<br>Mean calculated to be 108950±59995.58 | | 15 |
| (Sanders-van Wijk, van Empel et al. 2015) | Switzerland, Germany, part of TIME-CHF study | Chronic heart failure, history of hospitalisation within last year, NT-BNP levels higher than double the normal limit | No patients with dyspnoea caused by reasons other than heart failure, valvular heart disease needing surgery, acute coronary syndrome within 10 days, angina pectoris due to document ischaemia, percutaneous coronary intervention within last month, coronary artery bypass graft surgery within last 3 months, life expectancy under 3 years for diseases other than cardiovascular | Median follow up of 540-543 days | Levels of ST2 and other biomarkers | All-cause mortality and hospitalisation for heart failure during follow up | 570 | HFrEF (N=458): 76±7<br><br>HFpEF (N=112): 80±7 | Observational | Serum ST2 | Blood collected at baseline<br><br>HFrEF (N=413): Median is 35700<br>Mean calculated to be 37833.33±19788.37<br><br>HFpEF (N=100): Median is 37600<br>Mean calculated to be 40266.67±19707.77 | | 10 |
| (Frioes, Lourenco et al. 2015) | Portugal | Acute heart failure (including both reduced and preserved | No patients with acute coronary syndrome, chronic renal function replacement therapy | 6 month follow up | Levels of ST2 and BNP | All-cause mortality and hospital admission | 195 | 74.8±12.3 | Observational | Serum ST2<br><br>ELISA R&D systems, | Blood collected at last day of hospitalisation<br><br>Patients received standard treatment, | | 12 |

| | | | | | | | | | | | | | |
|---|---|---|---|---|---|---|---|---|---|---|---|---|---|
| | | ejection fraction) | | | | for acute heart failure during follow up | | | | Abingdon UK<br><br>0.005 ng/ml sensitivity | discharge at doctor's discretion<br><br>HFpEF median is 30.5<br>Mean calculated to be 32.77±17.08<br><br>HFrEF median is 29.8<br>Mean calculated to be 30.6±13.96<br><br>Overall Median is 30.3<br>Mean calculated to be 31.4±15.16<br><br>Events (66 patients): Median is 33.6<br>Mean calculated to be 36.5±15.08<br><br>No events (129 patients): Median is 26.8<br>Mean calculated to be 27.63±12.22 | | |
| (Ruocco, Evangelista et al. 2019) | Italy | Acute heart failure with and without diabetes | No patients with respiratory dyspnoea due to bronchial asthma, COPD, interstitial lung disease. Also no patients with acute or recent myocardial ischaemia, severe renal insufficiency, neoplastic/haematological/immune diseases with systemic involvement. | 6 months follow up | Levels of ST2 and BNP | Hospitalisation and deaths during follow up | 121 | HFrEF: Median is 82<br><br>HFpEF: Median is 83 | Observational | Serum ST2<br><br>Presage ST2 | HFrEF: Median is 63000<br>Mean calculated to be 66333.33±62285.77<br><br>HFpEF: Median is 48000<br>Mean calculated to be 59666.67±52390.21 | | 13 |
| (Zhang, Meng et al. 2018) | China | Acute heart failure (446 ischaemic heart failure, 574 non-ischaemic heart failure) | No patients with acute coronary syndrome (including unstable angina pectoris and acute MI), cancer, acute pulmonary embolism, unclear primary aetiology of HF | 1 year follow up<br><br>Multi-HR ischaemic all-cause mortality per log sST2: 2.38 (1.64-3.47)<br><br>Multi-HR Non-ischaemic all-cause mortality per log sST2: 2.16 (1.7-2.76)<br><br>Adjustments: Age, sex, diabetes mellitus, systolic blood pressure, New York Heart Association functional class, left ventricular ejection fraction, hemoglobin, sodium, C-reactive protein, angiotensin-converting enzyme inhibitor/angiotensin receptor blocker treatment, -blockers and estimated | Levels of ST2 and other biomarkers | All-cause mortality during follow up | 1020 | 57.5±15.7 | Observational | Serum ST2<br><br>ELISA Critical Diagnostics | Blood collected within 12 hours of admission (also says obtained day after admission)<br><br>Median is 37700<br>Mean calculated to be 40066.67±22790.5<br><br>All patients received intravenous loop diuretics during first 24H of admission | | 13 |

| | | | | | | | | | | | | | |
|---|---|---|---|---|---|---|---|---|---|---|---|---|---|
| | | | | glomerular filtration rate (GFR) | | | | | | | | | |
| (Tang, Wu et al. 2016) | Multinational (in this substudy, mostly from N. America)<br><br>Analysis of ASCEND-HF trial, which looked at Nesiritide in decompensated heart failure | Acute decompensated heart failure | Not stated, refers to ASCEND-HF trial for criteria | 180 days follow up<br><br>Multi-HR all-cause mortality per log sST2: 1.35 (0.9-2.03)<br><br>Adjustments: ASCEND-HF (Acute Study of Clinical Effectiveness of Nesiritide in Decompensated Heart Failure) trial risk model according to endpoints, NT-proBNP<br><br>Uni-HR all-cause mortality per log sST2: 2.21 (1.57-3.13) | All-cause mortality during follow up | Levels of ST2 | 858 | 65.5±15.2 | Post-hoc | Serum ST2<br><br>Presage | Blood taken at baseline<br><br>Median is 71200<br>Mean calculated to be 76833.33±46707.15 | | 11 |
| (Jin, Wei et al. 2017) | China | Acute heart failure (including acute exacerbation of chronic heart failure) | No patients with malignancies, cognitive deficit, severe mental illness, uncontrolled systemic disease. | 1 year follow up<br><br>Multi-HR all-cause mortality per ln sST2: 1.542 (1.001-2.375)<br><br>Adjustments: age, sex, hypertension, diabetes mellitus, renal insufficiency, hemoglobin, serum sodium, ln-transformed NT-proBNP, and NYHA | Levels of ST2 and other biomarkers | All-cause mortality during follow up | 270 | 60.5±16.4 | Observational | Serum ST2<br><br>ELISA R&D systems, Abingdon UK<br><br>3.1 ng/ml sensitivity | Blood collected at admission or morning after<br><br>Median is 31760<br>Mean calculated to be 35476.67±28523.29<br><br>223 survivors, 47 deaths in 1 year<br><br>Survivors: Median is 29070<br>Mean calculated to be 31396.67±23101.33<br><br>Deaths: Median is 55640<br>Mean calculated to be 70086.67±71441.64 | | 13 |
| (van Vark, Lesman-Leegte et al. 2017) | Netherlands (part of TRIUMPH study) | Acute heart failure (including decompensated chronic heart failure) | No patients with HF caused by non-cardiac reason, severe valvular dysfunction without sustained left ventricular dysfunction, acute STEMI, scheduled for coronary revascularisation, severe renal failure | Median of 325 days follow up<br><br>Multi-HR MACE per 1 SD increase of log$_2$ scale sST2: 1.3 (1.08-1.56)<br><br>Multi-HR all-cause mortality per 1 SD increase of log$_2$ scale sST2: 1.43 (1.11-1.86)<br><br>Adjustments: Age, sex, systolic blood pressure, diabetes mellitus, left ventricular ejection fraction, previous hospitalization for HF during the last 6 months, ischemic heart failure, body | Levels of ST2 | All-cause mortality and readmission for heart failure during follow up | 475 | Median is 74<br>Mean calculated to be 73±11.15 | Observational | Serum ST2<br><br>Presage ST2 assay | Blood taken at admission, then days 2-4, discharge, until 12 months after discharge<br><br>Median is 71000<br>Mean calculated 73000±41640.52 | | 11 |

| | | | | | | | | | | | | | |
|---|---|---|---|---|---|---|---|---|---|---|---|---|---|
| | | | | mass index, estimated glomerular filtration rate, and baseline NT-proBNP<br><br>Uni-HR all-cause mortality per 1 SD $log_2$ sST2: 1.8 (1.41-2.29)<br><br>Uni-HR MACE per 1 SD $log_2$ sST2: 1.49 (1.26-1.77) | | | | | | | | | |
| (Pascual-Figal, Manzano-Fernandez et al. 2011) | Spain | Acute decompensated heart failure | Not stated | Followed up for median of 739 days<br><br>Multi-HR all-cause mortality per log sST2 (back transformed to show clinical relevance): 1.09 (1.03-1.15)<br><br>Adjustments: Sex, body mass index, and left ventricular ejection fraction<br><br>Uni-HR all-cause mortality per log sST2 (back transformed to show clinical relevance): 1.09 (1.04-1.13) | All-cause mortality during follow up | Levels of ST2 and other biomarkers | 107 | 72±13 | Observational | Serum ST2<br><br>Presage ST2 | Blood taken at admission<br><br>Median is 62960 Mean calculated to be 69853.33±49598.05<br><br>Data for survival seems incorrect, the median is lower than the 25th percentile | | 12 |
| (Pascual-Figal, Bayes-Genis et al. 2019) | Spain | Acute decompensated heart failure (including decompensated chronic HF) | Not stated | 1 year follow up<br><br>Multi-HR all-cause mortality per 1 SD of ln sST2: 1.39 (1.05-1.84)<br><br>Adjustments: Gender; Age; Body mass index; Diabetes mellitus; Cerebrovascular disease; Coronary disease; Prior myocardial infarction; Prior heart failure hospitalization; New York Heart Association class; Beta-blockers; Mineralocorticoid receptor antagonists; Systolic blood pressure; Diastolic blood pressure; Creatinine; Urea; Sodium; Potassium; N-terminal pro-B-Type natriuretic peptide; high sensitivity Troponin T; Left ventricular ejection | All-cause mortality during follow up | Levels of ST2 and other biomarkers | 316 | 71.8±11.7 | Observational | Serum ST2<br><br>Presage ST2 | Blood taken at admission<br><br>58130±38830 | | 11 |

| | | | | | | | | | | | | | |
|---|---|---|---|---|---|---|---|---|---|---|---|---|---|
| | | | | fraction; C-reactive protein.<br><br>Uni-HR all-cause mortality per 1 SD ln sST2: 1.56 (1.2-2.03) | | | | | | | | | |
| (Manzano-Fernandez, Mueller et al. 2011) | Multicentre, based on 3 similar previous study populations<br><br>Diagnostic accuracy of B type natriuretic peptide and amino terminal proBNP in the emergency diagnosis of heart failure<br><br>The N-terminal pro-BNP investigation of dyspnea in the emergency department (PRIDE) study<br><br>Complementary prognostic value of cystatin C, N-terminal pro-B-type natriuretic peptide and cardiac troponin T in patients with acute heart failure | Acutely decompensated heart failure | Not stated | 1 year follow up<br><br>Multi-HR all-cause mortality per log sST2 (back transformed to show clinical relevance): 1.23 (1.13-1.34)<br><br>Adjustments: Age, Body mass index, Systolic blood pressure, Diastolic blood pressure, Previous heart failure, New York Heart Association functional class, β Blocker, Angiotensin-converting enzyme inhibitor, Hemoglobin (g/dl), Leukocytes, Creatinine, Estimated glomerular filtration rate, Blood urea nitrogen, C-reactive protein, Plasma amino terminal B-type natriuretic peptide<br><br>Uni-HR all-cause mortality per log sST2 (back transformed to show clinical relevance): 1.74 (1.43-2.12) | Levels of ST2 | Mortality during follow up | 447 | 73±13 | Observational | Serum ST2<br><br>ELISA Medical and Biological Laboratories, Woburn | Measurements made using frozen blood samples<br><br>Median is 470<br>Mean calculated to be 563.33±490.86<br><br>117 died: Median is 800<br>Mean calculated to be 1016.67±1058.36<br><br>330 survivors: Median is 380<br>Mean calculated to be 446.67±357.4 | | 10 |
| (Jackson, Haig et al. 2016) | Scotland, multicentre<br><br>Profile of microvolt T-wave alternans testing in 1003 patients hospitalized with heart failure | Decompensated heart failure (but studied 1 month post-hospitalisation so considered stable) | No patients with primary presentation with MI, concurrent systemic disease that reduces life expectancy | Mean follow up of 3.2±1.5 years<br><br>Multi-HR all-cause mortality per log sST2: 1.31 (1.04-1.65)<br><br>Adjustments: LVEF, BMI per unit decrease, Age, Sex, Diabetes, HF, NYHA, SBP, Peripheral edema, Previous MI, Current smoker, PAD, Lymphocytes, COPD, Creatinine, Hemoglobin, Bilirubin, log BNP<br><br>Uni-HR all-cause mortality per log sST2: 1.53 (1.23-1.9) | All-cause mortality during follow up | Levels of ST2 and other biomarkers | 628 | 70.8±10.6 | Observational | Serum ST2<br><br>ELISA R&D systems, Abingdon UK<br><br>0.1 ng/ml sensitivity | Blood taken at 1 month post hospitalisation<br><br>Median is 19900<br>Mean calculated to be 20300±7727.46 | | 11 |

| | | | | | | | | | | | | | |
|---|---|---|---|---|---|---|---|---|---|---|---|---|---|
| (Sugano, Seo et al. 2019) | Japan | Acute decompensated heart failure (HFpEF) | No patients with malignancy, acute coronary syndrome, severe valvular heart disease, hypertrophic cardiomyopathy, end stage renal disease | Median follow up of 445 days<br><br>Multi-HR all-cause mortality per log sST2 (back transformed for clinical significance): 1.02 (1.009-1.04)<br><br>Adjustments: Age, BMI, glomerular filtration rate, Haemoglobin, BNP, CRP, Noradrenaline<br><br>Uni-HR all-cause mortality per log sST2 (back transformed for clinical significance): 1.02 (1.01-1.03) | Levels of ST2 | Cardiovascular death, all-cause mortality during follow up | 191 | 76.4±11.9 | Observational | Serum ST2<br><br>ELISA R&D systems | Blood taken before discharge in stable condition<br><br>Median is 18<br>Mean calculated to be 18.7±10.68 | | 11 |
| (Pacho, Domingo et al. 2018) | Part of STOP-HF cohort | Acute decompensated heart failure | Not stated | 1 year follow up<br><br>Multi-HR MACE per 1 SD of log transformed sST2: 1.41 (1.21-1.63)<br><br>Multi-HR all-cause mortality per 1 SD of log transformed sST2: 1.45 (1.21-1.74)<br><br>Adjustments: Age, sex, NYHA, Charlson comborbidity index, Barthel index, urea, Hb, NT-proBNP, CA125, Hs-Tnl<br><br>Uni-HR all-cause mortality per 1 SD of log sST2: 1.86 (1.59-2.18)<br><br>Uni-HR MACE per 1 SD log sST2: 1.63 (1.44-1.85) | All-cause mortality and hospitalisation for heart failure during follow up | Levels of ST2 and other biomarkers | 522 | 82.1±8.7 | Observational | Serum ST2<br><br>Presage ST2 | Blood taken 4.9±2 days after discharge<br><br>Median is 42700<br>Mean calculated to be 45800±24457.04 | | 10 |
| (Wang, Yang et al. 2016) | Taiwan (China) | Acute heart failure and decompensated chronic HF | No patients with disorder other than HF that could compromise survival for next 6 months, bedridden for more than 3 months, dialysis in last 2 weeks, severe coronary artery disease without complete revascularisation therapy, pregnancy | Follow up of 3.7±1.3 years<br><br>Uni-HR all-cause mortality per log sST2: 2.652 (1.473-4.773)<br><br>Uni-HR MACE per log sST2: 2.156 (1.489-4.249) | Levels of ST2 and Gal-3 | Mortality and hospitalisation for heart failure during follow up | 344 (288 acute heart failure) | 60.6±13.4 | Observational | Serum ST2<br><br>Presage ST2 assay Critical Diagnostics<br><br>1.8 ng/ml sensitivity | Blood collected at enrolment<br><br>48300±46100 | | 12 |
| (Llibre, Zamora et al. 2016) | Spain | Acute heart failure | Not stated | 1 year follow up<br><br>Uni-HR all-cause mortality per 1 SD log sST2: 1.58 (1.12-2.24)<br><br>Uni-HR MACE per 1 SD log sST2: 1.44 (1.08-1.92) | All-cause mortality and hospitalisation for heart failure during follow up | Levels of ST2 | 182 | 69.2±12.8 | Observational | Serum ST2<br><br>Presage ST2 | Blood taken at admission<br><br>Median is 59100<br>Mean calculated to be 62800±41174.49 | | 11 |

| | | | | | | | | | | | | | |
|---|---|---|---|---|---|---|---|---|---|---|---|---|---|
| (Breidthardt, Balmelli et al. 2013) | Switzerland | Acute heart failure | No patients undergoing haemodialysis | Median of 368 days follow up | Levels of ST2 | Mortality during follow up | 207 | Median is 80<br>Mean calculated to be 79.67±8.21 | Observational | Serum ST2<br><br>Presage ST2 | Blood taken at presentation<br><br>Median is 78000<br>Mean calculated to be 81666.67±55990.85<br><br>138 Survivors:<br>Median is 65000<br>Mean calculated to be 67666.67±41954.26<br><br>Died: Median is 120000<br>Mean calculated to be 128666.67±81774.05 | | 12 |
| (Ky, French et al. 2011) | Multicentre, USA (Penn Heart Failure Study)<br><br>Comparison of MMP-9 and BNP as clinical biomarkers in chronic heart failure<br><br>Neuregulin-1beta is Associated with Disease Severity and Adverse Outcomes in Chronic Heart Failure | Chronic heart failure | No patients with non-cardiac condition resulting in mortality within 6 months | Median follow up of 2.8 years<br><br>Multi-HR MACE per $log_2$ sST2: 1.4 (1.2-1.7)<br><br>Adjustments: Age, gender, and race, cardiomyopathy etiology (ischemic versus nonischemic), tobacco use, body mass index, systolic blood pressure, creatinine, ejection fraction, biventricular pacemaker, cardioverter-defibrillator, ACE inhibitor/angiotensin receptor blocker, aldosterone antagonist, beta blocker therapy, and clinical site, NT-proBNP<br><br>Uni-HR MACE per $log_2$ sST2: 1.7 (1.5-1.9) | Levels of ST2 | All-cause mortality and cardiac transplantation | 1141 | 56.3±14 | Observational | Serum ST2<br><br>Presage ST2 | Blood taken at admission<br><br>Median is 27500<br>Mean calculated to be 30033.33±17665.53 | | 11 |
| (Wojtczak-Soska, Sakowicz et al. 2014) | Poland<br><br>Prognostic value of ST2 in short term follow up for chronic HF | Chronic systolic heart failure | No patients with NYHA 4, acute HF, acute coronary syndrome, inflammatory states, thyroid dysfunction | 1 year follow up | Composite endpoint is death or hospitalisation for HF during follow up | Levels of ST2 | 167 | 62.92±11.58 | Observational | Serum ST2<br><br>ELISA Medical and Biological Laboratories | Blood collected at admission<br><br>650±700<br><br>Composite endpoint (93 patients): 820±860<br><br>No composite endpoint (74 patients): 440±300 | | 11 |
| (Sobczak, Wojtczak-Soska et al. 2014) | Poland<br><br>Prognostic value of ST2 in chronic HF | Chronic heart failure | No patients with acute HF, acute coronary syndrome, autoimmune disease, inflammatory states | 1 year follow up | Composite endpoint is cardiova | Levels of ST2 | 145 | 62.16±11.25 | Observational | Serum ST2<br><br>ELISA Medical and | Blood taken at admission<br><br>Median is 515 | | 11 |

| | | | | | | | | | | | | | |
|---|---|---|---|---|---|---|---|---|---|---|---|---|---|
| | | | | | scular death, hospitalisation for HF exacerbation during follow up | | | | | Biological Laboratories<br><br>0.032 ng/ml sensitivity | Mean calculated to be 740.33±849.14<br><br>For composite endpoint: Median is 670<br>Mean calculated to be 905.33±1081.9<br><br>For no composite endpoint: Median is 377<br>Mean calculated to be 550.33±551.02 | | |
| (Rienstra, Yin et al. 2014) | USA, part of Framingham cohort, from the 6th examination | Community population | No atrial fibrillation | 10 year follow up<br><br>Muli-HR AF risk per 1 SD ln sST2: 1 (0.87-1.15)<br><br>Adjustments: Sex, age, smoking status, height, weight, systolic and diastolic blood pressure, hypertension treatment, diabetes status, heart failure and myocardial infarction, ln CRP, ln BNP | Levels of ST2 | Occurrence of atrial fibrillation during follow up | 3217 | Controls: 59±10 | Observational | Serum ST2<br><br>Presage ST2 Critical Diagnostics<br><br>2 ng/ml sensitivity | | Median is 20800<br>Mean calculated to be 21066.67±6823.06 | 14 |
| (Eggers, Kempf et al. 2016) | Sweden, part of PIVUS cohort | Community population | No cardiovascular disease | 10 years follow up<br><br>Multi-HR all-cause mortality per 1 SD ln sST2: 0.95 (0.81-1.12)<br><br>Multi-HR CV events per 1 SD ln sST2: 0.95 (0.81-1.12)<br><br>Adjustments: Sex, hypertension, diabetes, HDL cholesterol, LDL cholesterol, current smoking, body mass index, previous CV disease (if applicable), CRP, eGFR and each investigated biomarker | Levels of ST2 | All-cause mortality and cardiovascular events during follow up | 864 | Controls: 70 | Observational | Serum ST2<br><br>Presage ST2 assay Critical Diagnostics | | Baseline<br><br>Median is 21300<br>Mean calculated to be 21633.33±6534.47 | 15 |
| (AbouEzzeddine, McKie et al. 2016) | USA, part of Olmsted country cohort | Community population | No HF or systolic dysfunction | Median of 12 years follow up<br><br>Multi-HR HF risk per 1 SD ln sST2: 1.19 (1.02-1.4)<br><br>Adjustments: Age, sex, BMI, eGFR, hypertension, systolic blood pressure, CAD and diabetes mellitus<br><br>Multi-HR MACE per 1 SD ln sST2: 1.09 (0.97-1.21) | Levels of ST2 and other biomarkers | Occurrence of heart failure and MACE during follow up | 1824 | Median is 61<br>Mean calculated to be 61.33±12.61 | Observational | Serum ST2 | Baseline<br><br>Median is 26200<br>Mean calculated to be 26400±9199.48 | | 14 |

| | | | | | | | | | | | | | |
|---|---|---|---|---|---|---|---|---|---|---|---|---|---|
| | | | | Adjustments: Age, sex, BMI, eGFR, hypertension, systolic blood pressure, CAD, diabetes mellitus, total cholesterol, HDL and smoking history<br><br>Uni-HR HF risk per 1 SD ln sST2: 1.26 (1.07-1.48) | | | | | | | | | |
| (Parikh, Seliger et al. 2016) | USA<br><br>Part of Cardiovascular Health Study (CHS) | Community population | No patients with past or current HF | Median of 13.7 years follow up<br><br>Multi-HR HF risk per ln sST2: 1.12 (0.93-1.35)<br><br>Multi-HR CV death per ln sST2: 1.18 (0.95-1.48)<br><br>Adjustments: demographics and history of coronary heart disease, smoking status, systolic blood pressure, heart rate, serum glucose, creatinine, albumin levels, and left ventricular hypertrophy by electrocardiogram, NTproBNP, hsTnT<br><br>Uni-HR HF risk per ln sST2: 2.06 (1.75-2.43) | Levels of ST2 | Occurrence of heart failure and cardiovascular death during follow up | 3915 | 72.7±5.5 (no heart failure cohort) | Observational, post-hoc | Serum ST2<br><br>Presage ST2 | | Blood collected at initial admission in the 1990s<br><br>Median is 23500 Mean calculated to be 57700±24283.54 | 14 |
| (Hughes, Appelbaum et al. 2014) | Finland (FINRISK97 cohort, selected from national register) | Community population | Not stated | Mean follow up of 15 years<br><br>Multi-HR all-cause mortality per 1 SD ln sST2: 1.09 (1.01-1.19)<br><br>Multi-HR MACE per 1 SD ln sST2: 1.02 (0.96-1.09)<br><br>Multi-HR CVD per 1 SD ln sST2: 1.01 (0.94-1.08)<br><br>Multi-HR CHD per 1 SD ln sST2: 1.004 (0.92-1.09)<br><br>Multi-HR MI per 1 SD ln sST2: 0.97 (0.86-1.1)<br><br>Adjustments: Framingham risk factors (HDL and nonHDL cholesterol, systolic blood | Levels of ST2 and other biomarkers | Occurrence of cardiovascular event and MACE during follow up | 8444 | 48.79±22.08 (control) | Observational | Serum ST2<br><br>Presage ST2<br><br>2.35 ng/ml sensitivity | | Blood taken at enrolment, in 1997<br><br>890 values were missing, data presented is imputed, as imputed and complete case analysis were similar<br><br>Median is 26950 Mean could not be calculated, as IQR provided as a single figure rather than quartiles. | 13 |

| | | | | | | | | | | | | | |
|---|---|---|---|---|---|---|---|---|---|---|---|---|---|
| | | | | pressure (BP), smoking, prevalent diabetes) and geographic area, r NT-proBNP (N-terminal Pro-Brain Natriuretic Peptide), eGFR (estimated glomerular filtration rate) and prevalent valvular heart disease<br><br>Multi-HR HF risk per 1 SD ln sST2: 1.06 (0.96-1.16)<br><br>Adjustments: Framingham risk factors (HDL and nonHDL cholesterol, systolic blood pressure (BP), smoking, prevalent diabetes) and geographic area, r NT-proBNP (N-terminal Pro-Brain Natriuretic Peptide), eGFR (estimated glomerular filtration rate) and prevalent valvular heart disease, hsTroponin I | | | | | | | | | |
| (Andersson, Preis et al. 2015) | USA (Framingham 6th cycle) | Community population | No patients with prevalent stroke, dementia, neurological conditions | Follow up over mean of 11.8±3 years | Levels of ST2 and GDF-15 | Occurrence of stroke during follow up | 3374 | 59±9.7 | Observational | Serum ST2<br><br>Presage ST2 | | Median ST2 of controls is 20900<br>Mean calculated to be 21166.67±6971.24 | 14 |
| (Alkhiary, Abdalaal et al. 2015) | Saudi Arabia | Atrial fibrillation (persistent) | No patients with congenital heart disease, ACS, cardiomyopathy, concomitant valvular heart disease, previous cardiac surgery, heart failure, renal insufficiency, thyroid dysfunction, chronic inflammation, autoimmune disease, sepsis, malignancy, liver disease, haematological disease, bronchial asthma | None | Levels of IL-33 and ST2 | None | 160 | 60.2±6.4 | Case control | Serum IL-33 RayBio Human IL-33 ELISA kit<br><br>Serum ST2 ELISA RayBio<br><br>Minimum detection of 2pg/ml for both | Blood taken when sinus heart rate measured<br><br>IL-33: Median is 155.2<br>Mean calculated to be 148.63±40.9<br><br>ST2: 970.5±98.6 | IL-33: Median is 92.6<br>Mean calculated to be 96.93±34.39<br><br>ST2: 361.4±44.8 | 15 |
| (Ma, Yuan et al. 2018) | China | Atrial fibrillation | No patients with cancer, acute infection, immunological diseases | None | Levels of ST2 in AF patients | None | 254 | 61±11 | Case control | Serum ST2<br><br>ELISA (Presage ST2 Critical Diagnostics)<br><br>2 ng/ml sensitivity | Blood taken at admission<br><br>Median is 21690<br>Mean calculated to be 22733.33±10030.83 | Median is 17040<br>Mean calculated to be 17650±6038.68 | 13 |
| (Chen, Qu et al. 2018) | China | Non-valvular atrial fibrillation | No patients with acute MI, stroke, congenital heart disease, significant HF, chronic pulmonary heart disease, dilated or hypertrophic cardiomyopathy, inflammatory | Median follow up of 6 months | Levels of ST2 in AF patients | Occurrence of HF during follow up | 290 | Paroxysmal atrial fibrillation: 65.8±10.9<br><br>Persistent atrial | Case control | Serum ST2<br><br>ELISA by Boyun | Blood taken at admission<br><br>Paroxysmal atrial fibrillation: Median is 276.4 | Median is 222.4<br>Mean calculated to be 225.63±112.98 | 13 |

| | | | | | | | | | | | | | |
|---|---|---|---|---|---|---|---|---|---|---|---|---|---|
| | | | states, autoimmune disease, thyroid dysfunction | | | | | fibrillation:<br>65.4±9.9 | | | Mean calculated to be 288.77±156.46<br><br>Persistent atrial fibrillation: Median is 285<br>Mean calculated to be 303.77±115.06<br><br>Overall ST2 is 297.13±134.75 | | |
| (Wolcott, Batra et al. 2017) | USA | Acute ischaemic stroke | No patients without baseline plasma samples | 90 days | Levels of ST2 | Mortality and favourable/ unfavourable outcomes during follow up | 646 | 69±15 | Observational | Serum ST2<br><br>Presage ST2<br><br>3.1 ng/ml sensitivity | Plasma samples collected at 7.1±3.3 hours after stroke.<br><br>Median ST2 level is 35000<br>Mean calculated to be 36833.33±17905.73<br><br>At 48H post stroke, 210 patients had their ST2 levels measured again, median ST2 level is 37400<br><br>At 90 days, poor outcome patients had median ST2 of 38200, good outcome patients had 32600<br><br>Patients who died had median ST2 of 51000, patients who survived had 33700 | | 9 |
| (Jiang, Liu et al. 2018) | China | Acute ischaemic stroke | Not stated | None | Levels of IL-33 | None | 120 | 62.2±8.2 | Case control | Serum IL-33<br><br>ELISA Sen-Xiong Company | 283±54.8 (blood taken within 24H of event) | 165±25.6 | 11 |
| (Liu, Xing et al. 2014) | China | Acute cerebral infarction | None had liver/kidney dysfunction, heart failure, severe infection, malignancy, potential infection symptoms, surgical/trauma history, aspirin/statin/anti-hypertensive drug consumption within 2 weeks | None | Levels of IL-33 | None | 90 | 51.2±6.8 | Case control | Serum IL-33<br><br>R&D systems | 22 patients Large infarct (>10cm$^3$): 447.94±274.65<br>20 patients Medium infarct (5-10cm$^3$): 281.74±105.36<br>20 patients Small infarct (<5cm$^3$): 176.56±100.27<br><br>Overall mean and pooled standard deviation: 306.79±213.33<br><br>Blood taken within 48H of event | 186.71±89.69 | 12 |
| (Qian, Yuanshao et al. 2016) | China | Acute ischaemic stroke | No patients with heart failure, surgery/trauma within 2 months, renal dysfunction, liver insufficiency, serious infections, Th2 related diseases such as asthma/dermatitis/anaphylaxis | 3 months follow up | Levels of IL-33 | Barthel Index score during follow up | 287 | 61.2±10.4 | Case control | Serum IL-33<br><br>ELISA Boyun Biotech Shanghai | Median is 57.68 (measured the morning after admission)<br><br>Using (Wan, Wang et al. 2014), mean | Median is 47.48<br><br>Mean calculated to be 46.64±11.40 | 12 |

| | | | | | | | | | | | | | |
|---|---|---|---|---|---|---|---|---|---|---|---|---|---|
| | | | | | | | | | | 0.5 ng/L sensitivity | calculated to be 59.79±23.73<br><br>Median is 62.53 for favourable outcome, mean calculated to be 63.69±25.57<br><br>Median is 49.83 for unfavourable outcome, mean calculated to be 49.08±19.17<br><br>Small infarct (<5cm$^3$): Median is 59.36 (134 patients)<br>Large infarct: 55.72 (41 patients)<br><br>Mild stroke is NIHSS score of <6, moderate to severe stroke is ≥6. For mild stroke (153 patients), median IL-33 is 60.32. For moderate to severe stroke (53 patients), median IL-33 is 52.77 | | |
| (Chen, Lin et al. 2018) | China | Acute ischaemic stroke | No patients with cerebral haemorrhage, subdural haematoma, intracranial space occupying lesions, cerebrovascular damage caused by trauma, heart failure, malignancy, autoimmune disease, severe infection, pregnancy. | None | Levels of IL-33 and ST2 | MRI scans for infarct volume | 190 | 67.3±10.9 | Case control | Serum IL-33 and ST2<br><br>Westang Biotech ELISA, Shanghai<br><br>15 pg/ml sensitivity for IL-33<br><br>30 pg/ml sensitivity for ST2 | Measurements made within 48H of symptoms<br><br>Median IL-33 is 29.8<br>Mean calculated to be 30.4±22.23<br><br>Median ST2 is 103<br>Mean calculated to be 102.33±61.58<br><br>Mild stroke is NIHSS score of <6, severe stroke is ≥6<br><br>Mild stroke (73 patients) had ST2 median of 820, severe stroke (39 patients) had ST2 median of 118 | Median IL-33 is 26.7<br>Mean calculated to be 27.63±22.06<br>Median ST2 is 27<br>Mean calculated to be 28±18.88 | 12 |
| (Stankovic, Ljujic et al. 2019) | Serbia | Patients recruited before carotid endarterectomy | Not stated | None | Levels of IL-33 and ST2 | None | 221 | ~60s | Case control | Serum IL-33 and ST2<br><br>ELISA R&D systems<br><br>0.069-1.51 pg/ml sensitivity for IL-33<br><br>2.45-13.5 pg/ml sensitivity for ST2 | Blood taken before surgery<br><br>IL-33: 35.86±7.93<br><br>ST2: 183±8.03 | IL-33: 12.29±1.8<br><br>ST2: 122.31±15.89 | 10 |

| | | | | | | | | | | | | | |
|---|---|---|---|---|---|---|---|---|---|---|---|---|---|
| (Li, Wang et al. 2019) | China | Acute ischaemic stroke | No patients with hydrocephalus, dementia, traumatic brain injury, Parkinson's, schizophrenia, active infection, haematological disease, history of cancer, liver/renal insufficiency, autoimmune disease | 1 year | Levels of IL-33 | Barthel Index score during follow up | 259 | 63.14±10.02 | Observational | Serum IL-33 | Blood taken within 24H of admission.<br><br>58760±20670<br><br>Favourable outcome patients had 62450±20500 after admission, unfavourable outcomes had 51580±19160 after admission.<br><br>Infarct considered small if less than 5 $cm^3$. The infarct volume of all patients was small.<br><br>Follow up after 2.17±0.57 years, 35 patients (13.5%) had recurrent ischaemic stroke, 224 didn't. Patients with recurrent stroke had IL-33 levels of 50680±17260 after admission, those that didn't had 60060±20910 | | 11 |
| (Dieplinger, Bocksruck 2017) | Austria | Acute ischaemic stroke | No patients with transient ischemic attack, non-stroke, haemorrhagic stroke. Also no patients without blood taken with 24H of admission. | 90 days follow up | All-cause mortality | Functional outcome | 721 | Median of 76<br>Mean calculated to be 75.33±13.37 | Observational | ST2<br><br>Presage ST2 | Overall median is 35000<br>Mean calculated to be 37666.67±16341.64<br><br>Deceased (81 patients): Median is 55000<br>Mean calculated to be 70333.33±63402.42<br><br>Survivors (640 patients): Median is 34000<br>Mean calculated to be 35666.67±14116.85 | | 13 |
| (Yin, Cao et al. 2019) | China | Essential hypertension (same as primary hypertension) | No patients with secondary hypertension, coronary artery disease, heart failure, stroke, valvular heart disease, collagen disease, diabetes, advanced liver disease, renal failure, malignancy, sepsis, inflammatory disease, recent trauma, mental disorder, autoimmune disease | None | Levels of IL-33 and ST2 | None | 472 | 54.29±9.31 | Case control | Serum IL-33 and ST2<br><br>IL-33: Human Pre-Mixed Multi—nalyte kit R&D systems<br><br>ST2: DST200 | Does not say when blood is collected, assumed to be on hospital visit.<br><br>IL-33 median was 108340<br>Mean calculated to be 107660±18726.08<br><br>ST2 median was 663840 | IL-33 median was 109650<br>Mean calculated to be 109720±16289.72<br><br>ST2 median was 549050<br>Mean calculated to be 565530±219207.38 | 11 |

| | | | | | | | | | | | | | |
|---|---|---|---|---|---|---|---|---|---|---|---|---|---|
| | | | | | | | | | | R&D systems | Mean calculated to be 665700±227853.9<br><br>Systolic pressure: 153.13±13.38mmHg<br><br>Diastolic pressure: 92.61±10.32mmHg | | |
| (Ates, Ozkayar et al. 2016) | Turkey | Primary hypertension | No patients with cardiovascular/cerebrovascular/ peripheral artery disease, diabetes, kidney disease, liver disease, infection, rheumatic inflammatory disease | None | Levels of IL-33 and ST2 | Carotid intima media thickness | 172 | 46±12 | Case control | Serum IL-33 and ST2<br><br>IL-33: ELISA Affymetix Company Vienna, 0.2 pg/L sensitivity<br><br>ST2: ELISA RayBiotech, 2 pg/L sensitivity | Blood taken at admission, after 8H fasting<br><br>IL-33 level is 0.0025±0.0002<br><br>ST2 median level is 0.08 (could not calculate mean) | IL-33 level is 0.0026±0.0003<br><br>ST2 median level is 0.046 (could not calculate mean) | 10 |
| (Yavuzer, Cengiz et al. 2016) | Turkey | Hypertension (untreated) | No patients with metabolic syndrome, diabetes, atherosclerosis, endocrine disease, malignancy, connective tissue disease, on blood pressure medication within 12 months | None | Levels of IL-33 | None | 66 | 46.3±5.8 | Case control | Serum IL-33<br><br>ELISA eBioscience | 5.85±1.02 | 5.34±0.67 | 11 |
